# Ascorbic Acid Regulates the Immunity, Anti-Oxidation and Apoptosis in Abalone *Haliotis discus hannai* Ino

**DOI:** 10.3390/antiox10091449

**Published:** 2021-09-13

**Authors:** Kai Luo, Xinxin Li, Liu Wang, Wanxiu Rao, Yang Wu, Yue Liu, Mingzhu Pan, Dong Huang, Wenbing Zhang, Kangsen Mai

**Affiliations:** The Key Laboratory of Aquaculture Nutrition and Feeds, Ministry of Agriculture and Rural Affairs, The Key Laboratory of Mariculture, Ministry of Education, Ocean University of China, Qingdao 266003, China; luokai@stu.ouc.edu.cn (K.L.); lixinxin5014@stu.ouc.edu.cn (X.L.); 21180511100@stu.ouc.edu.cn (L.W.); raowanxiu@stu.ouc.edu.cn (W.R.); wuyang1651@stu.ouc.edu.cn (Y.W.); liuyue@stu.ouc.edu.cn (Y.L.); pmz@stu.ouc.edu.cn (M.P.); huangdong@stu.ouc.edu.cn (D.H.); kmai@ouc.edu.cn (K.M.)

**Keywords:** abalone, ascorbic acid, immune, anti-oxidation, apoptosis

## Abstract

The present study was conducted to investigate the roles of ascorbic acid (AA) in immune response, anti-oxidation and apoptosis in abalone (*Haliotis discus hannai* Ino). Seven semi-purified diets with graded levels of AA (0, 50, 100, 200, 500, 1000 and 5000 mg/kg) were fed to abalone (initial weight: 12.01 ± 0.001 g, initial shell length: 48.44 ± 0.069 mm) for 100 days. The survival, weight gain rate and daily increment in shell length were not affected by dietary AA. The AA content in the gill, muscle and digestive glands of abalone was significantly increased by dietary AA. In terms of immunity, dietary AA significantly improved the total hemocyte count, respiratory burst and phagocytic activity in hemolymph, and lysozyme activity in cell-free hemolymph (CFH). In the digestive gland, the TLR-MyD88-dependent and TLR-MyD88-independent signaling pathways were suppressed by dietary AA supplementation. The mRNA levels of *β-defensin* and *arginase-I* in the digestive gland were significantly increased by dietary AA. In the gill, only the TLR-MyD88-dependent signaling pathway was depressed by dietary AA to reduce inflammation in abalone. The level of *mytimacin 6* in the gill was significantly upregulated by dietary AA. After *Vibrio parahaemolyticus* infection, the TLR signaling pathway in the digestive gland was suppressed by dietary AA, which reduced inflammation in the abalone. In terms of anti-oxidation, superoxide dismutase, glutathione peroxidase and catalase activities, as well as total anti-oxidative capacity and reduced glutathione content in CFH, were all significantly upregulated. The malondialdehyde content was significantly downregulated by dietary AA. The anti-oxidative capacity was improved by triggering the Keap1-Nrf2 pathway in abalone. In terms of apoptosis, dietary AA could enhance the anti-apoptosis ability via the JNK-Bcl-2/Bax signaling cascade in abalone. To conclude, dietary AA was involved in regulating immunity, anti-oxidation and apoptosis in abalone.

## 1. Introduction

Ascorbic acid (AA), also known as vitamin C, is known to be an essential micronutrient that serves as a cofactor associated with enhancing the activity of multiple human enzymes and contributes to immune response and development of the nervous system in humans [1,2]. Due to the limited ability of AA to be preserved in the organism, a regular and adequate intake is necessary to prevent AA deficiency. AA is required in trace amounts from exogenous sources for average growth, physiological activities, reproduction and health in most mammals [3,4,5]. Regarding immune functions, AA contributes to maintaining the immune barrier integrity and wound healing [1]. It can boost the function of phagocytes, such as improving their motility, chemotaxis, phagocytosis and microbial killing [6,7,8,9]. Previous studies have reported various immune effects of AA on B-cells, T-cells and NK cells in mammals [10,11,12]. Meanwhile, AA is also associated with apoptosis. It inhibits myocardial apoptosis by preventing Bax (BCL-2 associated X protein) and increases the ability of Bcl-2 (apoptosis regulator bcl-2) to inhibit cytochrome-C release from mitochondria into the cytoplasm and subsequently reduces caspase-3, which initiates apoptosis [13]. As a natural anti-oxidant, AA reduces reactive oxygen species (ROS) and reactive nitrogen species (RNS) by endowing them with electrons and preventing the oxidation of other compounds [14,15,16,17]. AA can also enable other anti-oxidative molecules, such as glutathione (GSH), β-carotene, urate and α-tocopherol, to regenerate from their respective free radicals [18,19].

In contrast to terrestrial animals, relatively few reports exist on immune response and apoptosis in aquatic animals. Dietary-appropriate amounts of AA can optimize the growth, health and stress resistance in aquatic animals [20]. Most studies on AA in aquatic animals focus on estimating the minimum AA requirements for maximum growth and formulating a least-cost diet. The AA requirements vary among species, size, diet and farming conditions. The recommended amount of dietary AA ranges from 10 to 10,000 mg/kg [21]. The minimum requirement of dietary AA to support the maximum growth rate (WG) was 53–186 mg/kg in Nile tilapia (*Oreochromis niloticus*), 1200 mg/kg in Pacific bluefin tuna (*Thunnus orientalis*) and 5000–1000 mg/kg in kuruma shrimp (*Marsupenaeus japonicas*) [22,23,24]. However, the WG or specific growth rate (SGR) was not significantly affected by dietary AA in some fish species, such as Siberian sturgeon (*Acipenser baerii*), red sea bream (*Pagrus major*) and Japanese eel (*Anguilla japonica*) [25,26,27].

Dietary AA supplementation has been shown to enhance immune functions, such as phagocytosis and lysozyme activity in many fish species [28,29,30]. Increased survival to pathogen exposure (e.g., bacteria and parasite) has been reported in Nile tilapia, Asian catfish (*Clarias batrachus*) and Pacific white shrimp (*Litopenaeus vannamei*) after dietary AA intake [31,32,33,34]. AA deficiency decreased lysozyme (LZM) activity, complement component 3 (C3) and C4 content in grass carp (*Ctenopharyngodon idella*) [35]. It can also reduce the levels of antimicrobial peptides, such as β-defensin and hepcidin, and anti-inflammatory cytokines including interleukin (IL) 4/13A, IL-10 and transforming growth factor (TGF) β1/2; while upregulating pro-inflammatory cytokines, including IL-1β and nuclear factor κB (NF-κB) in grass carp [35]. In addition, AA deficiency upregulated apoptotic protease activation factor-1, caspase-3 and caspase-7–9 to aggravate cell apoptosis in grass carp [35,36].

Abalone is one of the most important commercial species in the Archaeogastropoda order of mollusks. It is an important model animal for studying the ecological and developmental biology of gastropods (Mollusca) [37,38]. Among the farmed abalone species, the Pacific abalone (*H. discus hannai* Ino) is the most preferred farmed species in China. Mai reported that dietary supplementation of AA from 0 to 8000 mg/kg did not significantly affect the SGR or survival rate (SR) of juvenile abalone [39]. Wu et al. reported that dietary AA influenced the expression of genes related to anti-oxidative responses in the digestive gland of abalone to improve its stress resistance [40]. In addition, the outbreak of diseases could cause enormous economic losses to the abalone industry [41,42]. Mollusks lack adaptive immunity and rely on innate immunity [43]. Hence, the present study aimed to investigate the roles of AA in the regulation of immunity, anti-oxidation and apoptosis in abalone *H. discus hannai* and will provide scientific instruction for the healthy regulation of dietary formulation in abalone.

## 2. Materials and Methods

### 2.1. Ethical Statement

All animal care and handling procedures performed in the present study were approved by the Animal Care Committee of the Ocean University of China (Approval No. SPXY2020012).

### 2.2. Experimental Diet

The basal diet (AA0) was formulated from purified ingredients to contain approximately 30% of dietary protein and 3.5% of dietary lipids (Table 1). Casein (vitamin-free) and gelatin were used as the protein sources, and soybean oil and menhaden fish oil (1:1) were used as lipid sources, which could be sufficient to maintain optimal growth for *H. discus hannai* [44,45,46]. Graded levels of AA (0, 50, 100, 200, 500, 1000 and 5000 mg/kg) were added to the basal diet to formulate the seven experimental diets. The AA was added to the diets in the form of L-ascorbyl-2-monophosphate. These diets were designated as AA0, AA50, AA100, AA200, AA500, AA1000 and AA5000, respectively. The analyzed contents of AA in the diet were 0.00, 47.31, 78.25, 189.05, 451.73, 919.99, 4821.17 mg/kg, respectively. The method of high-performance liquid chromatography was used to analyze dietary AA content [40]. The dietary crude protein, crude lipid and ash were measured according to the standard methods of the Association of Analytical Chemists [47].

### 2.3. Leaching Test

The leaching of dietary AA was presented as the retention efficiency (RE). The experimental diets with three replicates were immersed into the seawater at 23.0 ± 0.5 °C. The immersed diets were taken out at an allotted time (1, 3, 6, 12 h, respectively) and were lyophilized for the analysis of AA content.

RE (%) = (AA retained in diet after immersion)/(AA contained in diet before immersion) × 100.

### 2.4. Feeding Trial

Abalone juveniles were obtained from a fishery company in Fuzhou (Fujian, China) and were acclimatized to laboratory conditions for two weeks, with the basal diet, in a recirculating water system with tanks (100 × 50 × 40 cm). After that, similarly-sized abalones (initial weight: 12.01 ± 0.001 g, initial shell length: 48.44 ± 0.069 mm) were randomly assigned to 21 tanks (45 abalones per tank). Every three tanks were considered one treatment. All tanks were kept in dim light with black plastic drapes. The abalones were hand-fed once daily at 17:00. The feces and uneaten diets were removed at 8:00 every morning. During the 100-day feeding trial, the water temperature was 23.0 ± 0.5 °C, salinity was 30–33‰, pH was 7.4–7.9 and dissolved oxygen was ≥7.0 mg/L.

### 2.5. Vibrio Parahaemolyticus Challenge Test

After the feeding trial, fifteen abalones from each tank were used for the challenge test with pathogenic *V. parahaemolyticus*. Abalones were challenged with 100 μL of *V. parahaemolyticus* (1.2 × 10^6^ cfu/mL) by muscle injection in vivo. The digestive gland was collected at 0, 6, 12, 24, 48 and 72 h, respectively, and stored at −80 °C for qPCR analysis.

### 2.6. Sample Collection

At the termination of the feeding trial, abalones were fasted for three days before being counted and weighed. All abalones were anesthetized with 5% ethyl alcohol before sampling. Hemolymph was collected from four abalones in each tank to analyze the total hemocyte count (THC), respiratory burst (RB) and phagocytic activity (PA). In order to analyze the enzyme activity in cell-free hemolymph (CFH), the hemolymph was collected from another ten abalones in each tank and centrifuged (3000× *g* at 4 °C) for 10 min; the CFH was stored at −80 °C until use. The digestive gland, gill, muscle and mantle were sampled from ten hemolymph-taken abalones per tank and stored at −80 °C until use.

### 2.7. Ascorbic Acid Content

The AA content in the CFH, muscle, mantle, gill and digestive gland was detected using a ferric-reducing ascorbate assay kit (BioVision, Milpitas, CA, USA). The CFH could be used directly to measure AA content according to the manufacturer’s instructions. The 0.1 g samples of muscle, mantle, gill and digestive gland were homogenized in 1 mL of pre-cooled distilled water. The AA content was measured by absorbance at 593 nm.

### 2.8. Immune Parameters in Hemolymph

The hemolymph was thoroughly mixed using an equal volume of pre-cooled anticoagulant (100 mmol/L EDTA Na_2_, 450 mmol/L NaCl, 10 mmol/L KCl, 10 mmol/L HEPES, pH 7.3, 850 mOs mol/kg) to dilute the hemolymph.

THC was counted using a hemocytometer under the microscope (CX31, OLYMPUS, Tokyo, Japan).

The reduction of nitroblue tetrazolium (NBT) in hemocytes, which can be represented by RB activity, was measured according to the methods of Anderson et al., with some modifications [48]. Briefly, the hemolymph was adjusted to 5 × 10^6^ cells/mL using anticoagulant and was centrifuged at 3000× *g* at 4 °C for 10 min. The cell precipitate was stained with 100 μL of 1 μg/mL PMA (phorbol myristate acetate, Sigma-Aldrich, Saint Louis, MO, USA) dissolved by DMSO (dimethyl sulfoxide, Solarbio, Beijing, China) and incubated at 37 °C for 30 min. After incubation, 100 μL of 0.3% NBT dissolved by Hank’s Balanced Salt Solution (Solarbio, Beijing, China) was added to the cells and incubated at 37 °C for 30 min before centrifugation (560× *g* at 4 °C for 10 min). The supernatant was gently removed, and the reaction was terminated using 200 μL methanol for 10 min. After that, the cell was washed three times with 70% (*v*/*v*) methanol before being air-dried. The formazan blue crystal was dissolved using 120 μL of 2 M KOH and 140 μL of DMSO. The optical density (OD) was read in a spectrophotometer at 630 nm against a KOH/DMSO blank.

The PA of hemolymph was determined following the previously described methods of Xue et al. [49]. Briefly, 50 μL of diluted hemolymph was placed on a glass slide and incubated at 25 °C for 20 min to promote adhesion. Then, 50 μL of yeast (*Saccharomyces cerevisiae*) with 1 × 10^6^ cells/mL was added to the hemocyte monolayer before incubation (25 °C for 30 min). The slide was gently washed twice with sterilized phosphate buffer saline (PBS) and was fixed with methanol for 5 min. After that, the slide was stained with Giemsa solution for 20 min. The cells were counted under an oil immersion lens. The phagocytic rate indicated phagocytic activity.

### 2.9. Biochemical Parameters in CFH

The anti-oxidative enzyme activities (such as SOD, catalase (CAT) and glutathione peroxidase (GPX)), total anti-oxidative capacity (T-AOC) and content of malondialdehyde (MDA) and GSH in CFH were determined using the commercial assay kits (Nanjing Jiancheng Bioengineering Institute, Catalog: T-AOC, A015-2-1; SOD, A001-3-2; CAT, A007-1-1; GSH, A006-2-1; GPX, A005-1-2; MDA, A003-1-2). The T-AOC was based on the generation of green ABTS (2, 2′-azino-bis (3-ethylbenzothiazoline-6-sulfonic acid)) radicals when the ABTS was oxidized. The SOD, GSH and GPX were measured by absorbances at 450 nm, 405 nm and 421 nm, respectively, using an ultraviolet spectrophotometer (Ultrospec 2100 pro, Biochrom, Holliston, MA, USA). The CAT and MDA were determined using the ammonium molybdate and TBA (thiobarbituric acid) methods [50,51].

The LZM activity and the content of C3 and C4 in the CFH were measured using commercial assay kits (Nanjing Jiancheng Bioengineering Institute, Nanjing, China. Catalog: LZM, A050-1-1; C3, E032-1-1; C4, E033-1-1). The LZM was measured by transmittance at 530 nm. The C3 and C4 were determined at 340 nm.

### 2.10. Total RNA Extraction and Quantitative Real-Time PCR

The total RNA from the digestive gland and gill was extracted using a tissue total RNA isolation kit (Vazyme, Nanjing, China) and TRIzol reagent (Invitrogen, Carlsbad, CA, USA) following the manufacturer’s instructions. The concentration and purity of extracted total RNA were determined using a Nanodrop 2000 (Thermo Fisher Scientific, Waltham, MA, USA). The complementary DNA (cDNA) was synthesized using a reverse transcription kit (PrimeScript^®^ RT reagent Kit with gDNA Eraser, Takara, Japan).

All primers were designed using Primer Premier 6.0 (Supplementary Table S1) and synthesized by Sangon Biotech (Shanghai, China). The amplification reaction was carried out in a total volume of 25 μL, containing 0.5 μL of forward and reverse primers, 12.5 μL of 2 × SYBR Green Realtime Master Mix (Vazyme), 1 μL of cDNA and 10.5 μL of sterilized ddH_2_O. The following thermocycling conditions were used to determine the expression profiles for each gene: 95 °C for 30 s; 40 cycles of 95 °C for 10 s and 60 °C for 30 s; with subsequent incubations at 95 °C for 15 s, 60 °C for 1 min and 95 °C for 1 s to detect fluorescence. The expression levels of the target gene were normalized to that of the combination of the two most stable reference genes (*β-actin* and *gapdh*), which were validated using geNorm and NormFinder [52,53]. The relative gene expressions were calculated using the 2^−ΔΔCT^ method [54].

### 2.11. Western Blot

The total protein of the digestive gland and gill was extracted using the RIPA (Solarbio) method [55] with protease and phosphatase inhibitor cocktails (Roche, Basel, Switzerland). According to the manual, the nuclear protein of the digestive gland and gill was extracted using a Nuclear and Cytoplasmic Protein Extraction Kit (Beyotime, Shanghai, China). Protein concentration was measured using the BCA Protein Quantification Kit (Vazyme, Nanjing, China), and all protein samples were diluted to an equivalent concentration of 1.5 μg/μL using a RIPA reagent. Protein samples from the digestive gland and gill were separated using SDS-PAGE and subsequently transferred to a 0.45 μm PVDF membrane. The PVDF was blocked using 5% nonfat powdered milk (Beyotime, Shanghai, China) in preparation of TBST at room temperature for 1 h. The membrane was washed three times with TBST before incubation using primary antibody overnight at 4 °C at 60 rpm. After that, the incubated membrane was washed three times with TBST and was incubated using goat anti-rabbit horseradish peroxidase-conjugated secondary antibody (Beyotime, Shanghai, China) for 1 h at room temperature. The PVDF membrane was visualized using an ECL chemiluminescence kit (Vazyme, Nanjing, China). The GAPDH (1:500, AB-P-R001, Goodhere Biotechnology, Hangzhou, China) and Lamin B (1:500, WL01775, Wanleibio, Shenyang, China) were used as reference proteins. The primary target antibodies were myeloid differentiation primary response 88 (MyD88, 1:500, WL02494, Wanleibio, Shenyang, China), c-Jun N-terminal kinase (JNK, 1:500, WL01295, Wanleibio, Shenyang, China), mature-IL-1β (1:500, WL00891, Wanleibio, Shenyang, China), NF-κB p65 (1:500, WL01980, Wanleibio, Shenyang, China), kelch-like ECH associating protein 1 (Keap1, 1:1000, WL03285, Wanleibio, Shenyang, China) and cleaved-caspase3 (1:500, WL02117, Wanleibio, Shenyang, China). The quantification of Western blot was calculated using ImageJ (Version 1.53, Wayne Rasband and contributors, National Institute of Health, Bethesda, MD, USA).

### 2.12. Calculations and Statistical Analysis

SPSS 24 software (IBM, Armonk, NY, USA) was used for statistical analyses. One-way ANOVA was applied with Tukey’s multiple range test for detecting statistical differences between these groups at the significance level of 0.05. All data were expressed as the mean ± SE (standard error).

The SR, WGR (weight gain rate) and DISL (daily increment in shell length) were calculated as follows:

SR (%) = (number of survived abalone/number of initial abalone) × 100;

WGR (%) = [(final weight (g) − initial weight (g))/initial weight (g)] × 100;

DISL (μm/day) = [final shell length (mm) − initial shell length(mm)]/days × 1000.

## 3. Results

### 3.1. Retention Efficiency of Ascorbic Acid in the Diet

The RE of dietary AA at different intervals immersed in seawater is shown in Supplementary Figure S1. In general, the RE of AA in the diet was decreased with the duration of immersion in seawater. During the feeding experiment, the diet stayed in the seawater for approximately 12 h. Therefore, the leaching trial lasted for 12 h in seawater under the same feeding conditions. After a 3 h immersion, the RE of AA in diets ranged from 95.25% to 97.43%. The RE of AA was maintained at more than 95% in the 6 h immersion. After immersing for 12 h, the RE of AA in AA50, AA100, AA200, AA500, AA1000 and AA5000 were 89.78%, 90.88%, 89.38%, 91.03%, 87.44% and 86.78%, respectively.

### 3.2. Growth Performance and Ascorbic Acid Distribution in Tissues

The SR and growth performance of abalone fed gradient levels of AA supplementation are shown in Table 2. The SR (%), WGR (%) and DISL (μm/day) were not significantly affected by dietary AA levels (*p* > 0.05). The SR varied between 80.74% and 88.15%, and the WGR and DISL ranged from 29.32% to 31.52% and 17.92 to 20.01 μm/day, respectively.

**Figure 1:**
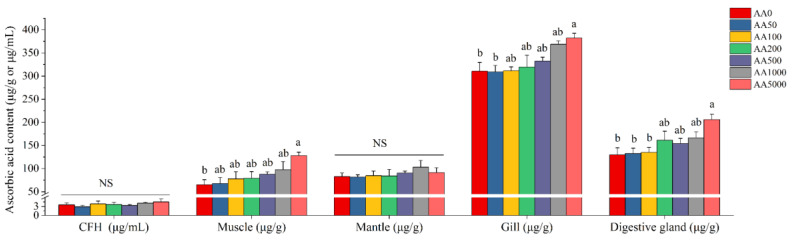
Ascorbic acid content in different tissues of abalone (*Haliotis discus hannai*) after a 100-day feeding trial. All data expressed as mean ± SE (*n* = 3). The different lowercase letters indicate significant differences (*p* < 0.05). NS: non-significant difference; CFH: cell-free hemolymph.

The AA distribution in tissues, including CFH, muscle, mantle, gill and digestive gland, is shown in Figure 1. No significant differences in AA content in CFH and mantle of abalone after dietary AA were observed (*p* > 0.05). AA content in CFH and mantle ranged from 2.89 μg/mL to 4.52 μg/mL and 81.63 μg/g to 103.08 μg/g, respectively. The AA contents of muscle, gill and digestive gland were significantly increased (*p* < 0.05) and reached their peak at the AA5000 group. The AA content in the muscle, gill and digestive gland was 64.88–128.20 μg/g, 308.98–382.19 μg/g and 130.08–206.09 μg/g, respectively.

### 3.3. Hemolymph Immune Parameters

The THC, RB and PA of hemolymph are presented in Table 3. The THC significantly increased with the increase of dietary AA, ranging from 1.07 × 10^7^–1.65 × 10^7^ cells/mL, and reached its peak at the AA1000 group.

The RB in the hemocyte of abalone was significantly increased and reached its highest level at the AA1000 group (0.35 OD_630_/10^7^ cells·mL^−1^). As the supplementation of dietary AA increased from 189.05 mg/kg to 919.99 mg/kg, the RB was significantly increased from 0.20 OD_630_/10^7^ cells·mL^−1^ to 0.35 OD_630_/10^7^ cells·mL^−1^ (*p* < 0.05). Dietary AA could affect the PA in hemocytes of abalone (*p* < 0.05). The PA of abalone in the AA1000 and AA5000 group had significantly higher than that in AA0, AA50 and AA100 groups and achieved its maximum (48.92%) in the AA5000 group.

The immune-related parameters in the CFH of abalone, including LZM, C3 and C4 content, are presented in Table 4. The LZM activity was significantly elevated (*p* < 0.05) and attained the highest levels in the AA1000 group, where its activity was 56.25 U/mL. The data did not reveal any significant effects for C3 content among all treatments (*p* > 0.05); however, the C3 content remained increasing with the supplementation of dietary AA. The C4 content was not significantly affected by dietary AA (*p* > 0.05).

### 3.4. Anti-Oxidative Parameters in CFH

The anti-oxidative enzyme activities in the CFH of abalone containing SOD, CAT, and GPX, as well as the T-AOC capacity and content of MDA and GSH are shown in Table 5. Dietary AA significantly influenced the activities of SOD, CAT and GPX, and the T-AOC and contents of GSH and MDA. The activities of SOD, CAT and GPX, and the T-AOC and GSH content were significantly upregulated (*p* < 0.05), while MDA content was significantly downregulated in abalone (*p* < 0.05). No significant differences in these anti-oxidative parameters in the CFH of abalone were observed between the AA1000 and AA5000 groups.

### 3.5. Expressions of Immune-Related Genes and Proteins in Abalone Digestive Gland and Gill

**Figure 2:**
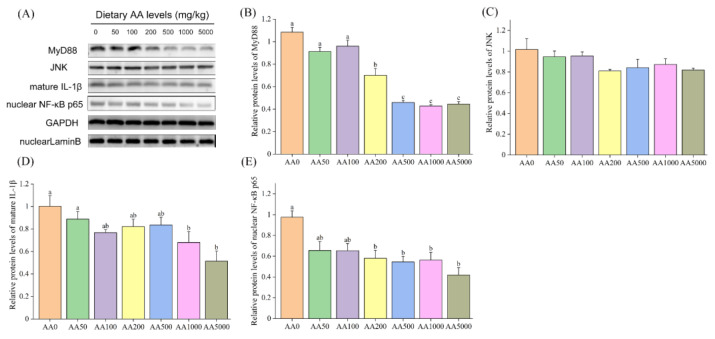
Protein expressions of MyD88, JNK, mature IL-1β and nuclear NF-κB p65 in the digestive gland of abalone (*Haliotis discus hannai)* after a 100-day feeding trial. (**A**) Representative Western Blot image of MyD88, JNK, mature IL-1β and nuclear NF-κB p65; (**B**–**E**) Protein expressions of MyD88, JNK and mature IL-1β and nuclear NF-κB p65. All data are expressed as the mean ± SE (*n* = 3). The different lowercase letters indicate significant differences (*p* < 0.05).

The mRNA levels of the pivotal genes in the toll-like receptor (TLR) signaling pathway in the digestive gland of abalone are shown in Table 6 and Table 7. The expression levels of *tlr2* and *tlr4* in the digestive gland were significantly decreased (*p* < 0.05) and had their lowest levels at the AA1000 and AA5000 groups, respectively (Table 6). The mRNA level of *tlr-a* was significantly upregulated (*p* < 0.05), and *tlr-b* was not affected by graded levels of dietary AA (*p* > 0.05). The expressions of *myd88*, *trif-related adaptor molecule* (*tram*), *interleukin 1 receptor-associated kinase 4* (*irak4*) and *tumor necrosis factor receptor-associated factor 6* (*traf6*) were significantly reduced after dietary AA supplementation (*p* < 0.05). The *traf4* was not influenced by the level of AA supplementation (*p* > 0.05). The mRNA levels of *iκb kinase α* (*ikkα*) and *nf-κb inhibitor α* (*iκbα*) in the digestive gland of abalone were not affected by dietary AA (*p* > 0.05) (Table 7). However, the expression of *p38 mitogen-activated protein kinase* (*p38 mapk*) and *jnk* were significantly downregulated with the increasing dietary AA (*p* < 0.05). Dietary AA could significantly reduce the expression levels of *nf-κb*, *activator protein 1* (*ap-1*) and *tumor necrosis factor α* (*tnf-α*) (*p* < 0.05), and the *interleukin 16* (*il 16*) level was not subject to dietary AA (*p* > 0.05). Protein expressions of MyD88, JNK, mature IL-1β and nuclear NF-κB p65 are shown in Figure 2. The protein levels of MyD88, mature IL-1β and nuclear NF-κB p65 in the digestive gland of abalone were significantly downregulated by dietary AA (*p* < 0.05). In contrast, the protein level of JNK was not influenced by AA (*p* > 0.05).

**Figure 3:**
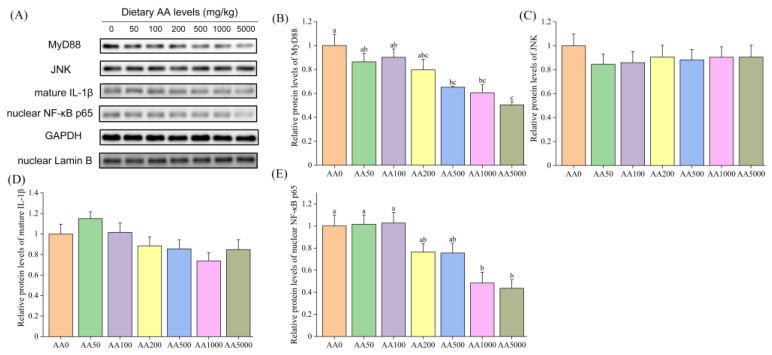
Protein expressions of MyD88, JNK, mature IL-1β and nuclear NF-κB p65 in the gill of abalone (*Haliotis discus hannai*) after a 100-day feeding trial. (**A**) Representative Western Blot image of MyD88, JNK, mature IL-1β and nuclear NF-κB p65; (**B**–**E**) Protein expressions of MyD88, JNK and mature IL-1β and nuclear NF-κB p65. All data were expressed as mean ± SE (*n* = 3). The different lowercase letters indicate significant differences (*p* < 0.05).

The mRNA levels of *tlr2*, *tlr4* and *tlr-b* in the gill of abalone were not significantly different (*p* > 0.05), and the *tlr-a* level was significantly reduced after graded levels of dietary AA (*p* < 0.05) (Table 8). Furthermore, the expression levels of *myd88*, *irak4* and *traf6* in the gill of abalone were significantly downregulated with the increasing level of dietary AA (*p* < 0.05). Instead, no significant differences in the expressions of *tram* and *traf4* were observed (*p* > 0.05). Dietary AA significantly downregulated the mRNA level of *ikkα* (*p* < 0.05), and the expressions of *iκbα*, *p38 mapk* and *jnk* in the gill were not affected by AA (*p* > 0.05) (Table 9). A One-way ANOVA revealed that the expression levels of *nf-κb*, *ap-1* and *tnf-α* were significantly reduced by dietary AA (*p* < 0.05), and no significant difference in *il 16* level was observed (*p* > 0.05). The protein levels of MyD88 and nuclear NF-κB p65 were significantly downregulated by graded levels of dietary AA (*p* < 0.05) (Figure 3). However, the protein expressions of JNK and mature IL-1β in the gill of abalone were not affected by dietary AA (*p* > 0.05).

Expressions of *β-defensin*, *mytimacin 6* and *arginase-I* in the digestive gland and gill of abalone are given in Table 10. The levels of *β-defensin* and *arginase-I* in the digestive gland were significantly increased after dietary AA (*p* < 0.05), whereas the expression of *mytimacin 6* was not affected by dietary AA (*p* > 0.05). In contrast, the mRNA levels of *β-defensin* and *arginase-I* in the gill were not significantly different, and the expression of *mytimacin 6* was significantly upregulated by dietary AA (*p* < 0.05).

### 3.6. Expressions of Immune-Related Genes in the Digestive Gland in Response to Vibrio Parahaemolyticus

**Figure 4:**
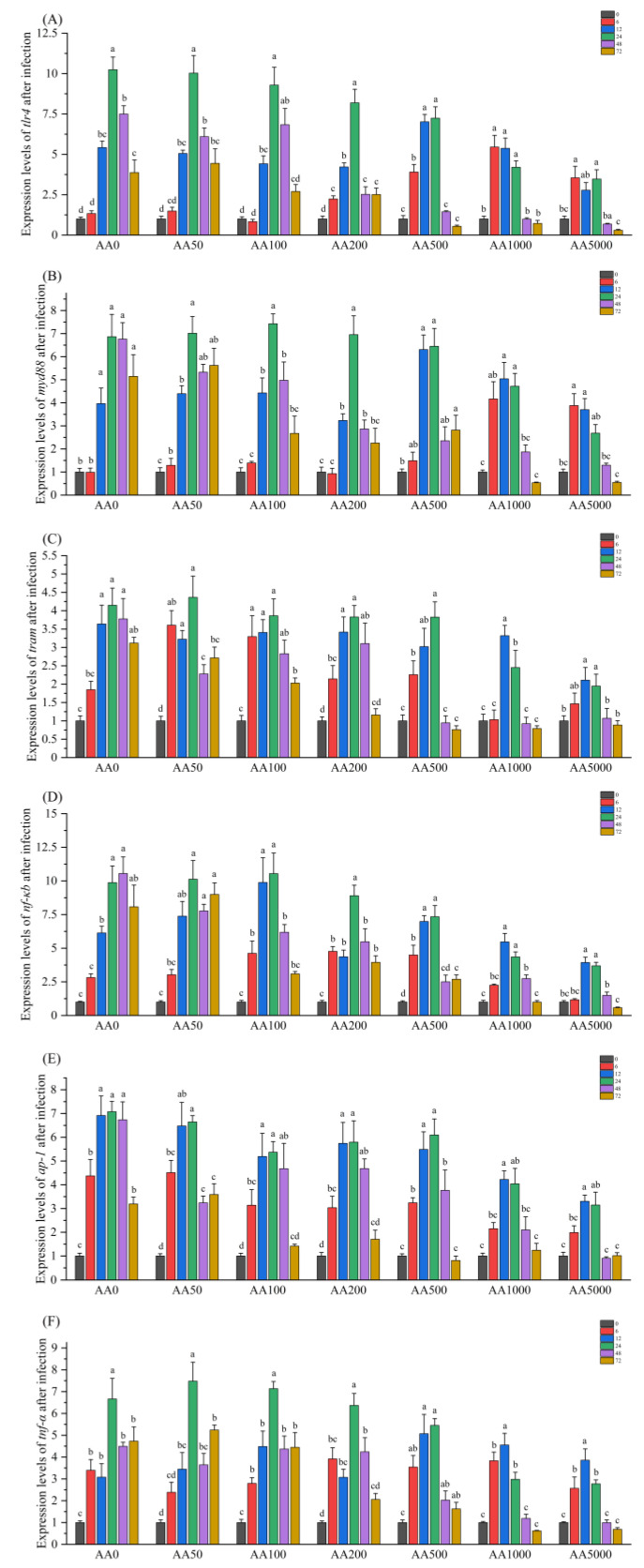
Expression levels of *tlr4*, *myd88*, *tram*, *nf-κb*, *ap-1* and *tnf-α* in the digestive gland of abalone (*Haliotis discus hannai*) in response to *Vibrio Parahemolyticus* after a 100-day feeding trial. (**A**) *tlr4*: *toll-like receptor 4*; (**B**) *myd88*: *myeloid differentiation primary response gene 88*; (**C**) *tram*: *trif-related adaptor molecule*; (**D**) *nf-κb*: *nuclear factor-κb*; (**E**) *ap-1*: *activator protein 1*; (**F**) *tnf-α*: *tumor necrosis factor α*. Gene expression levels are presented as the fold-change compared with the respective control group (AA0) (set to 1) at 0 h, 6 h, 12 h, 24 h, 48 h and 72 h post-infection. All data expressed as mean ± SE (*n* = 3). The different lowercase letters indicate significant differences (*p* < 0.05).

The expression levels of several critical genes in the TLR signaling pathway, containing *tlr4*, *myd88*, *tram*, *nf-κb*, *ap-1* and *tnf-α* in the digestive gland of abalone after *V. parahaemolyticus* infection are presented in Figure 4. The mRNA expressions of *tlr4*, *myd88*, *tram*, *nf-κb*, *ap-1* and *tnf-α* were significantly upregulated after *V. parahaemolyticus* administration (*p* < 0.05). The fold change of *tlr4* was highest in the AA0 group (10.24-fold) at 24 h post-infection, and subsequently, the highest level declined to 3.55-fold in the AA5000 group at 6 h. The mRNA level of *myd88* was significantly upregulated at 12–72 h in the AA0 group (6.86-fold) and at 6–12 h in the AA5000 group (3.88-fold) after *V. parahaemolyticus* infection. The expression of *tram* in the digestive gland of abalone after *V. parahaemolyticus* infection was significantly increased from 12 h to 72 h in the AA0 group and from 12 h to 24 h in the AA5000 group. The fold change of *nf-κb* reached its highest level at 48 h in the AA0 group (10.55-fold), followed by a decrease in the highest level at 12 h in the AA5000 group (3.93-fold change). The expression level of *ap-1* was significantly upregulated from 6 h to 72 h in the AA0 and AA50 group before recovering to normal level at 72 h after a continuous AA supplementation. The fold change of *tnf-α* reached its peak at 24 h after *V. parahaemolyticus* infection in AA0, AA50, AA100, AA200 and AA500 groups, and had its highest level at 12 h in the AA1000 and AA5000 groups.

**Figure 5:**
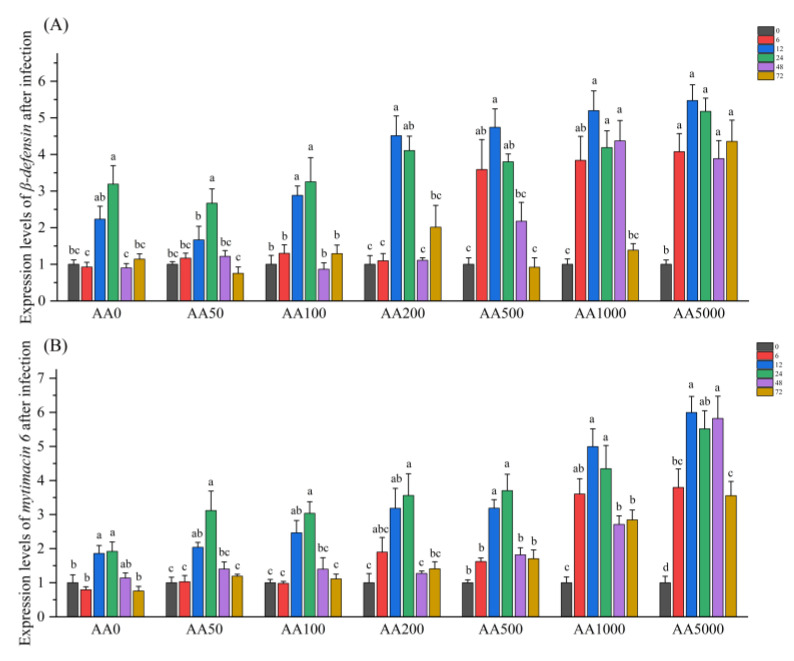
Expression levels of *β-defensin* and *mytimacin 6* in the digestive gland of abalone (*Haliotis discus hannai*) in response to *Vibrio Parahemolyticus* after a 100-day feeding trial. (**A**) *β-defensin*; (**B**) *mytimacin 6*. Gene expression levels are presented as the fold-change compared with the respective control group (AA0) (set to 1) at 0 h, 6 h, 12 h, 24 h, 48 h and 72 h post-infection. All data expressed as mean ± SE (*n* = 3). The different lowercase letters indicate significant differences (*p* < 0.05).

The results of the levels of *β-defensin* and *mytimacin 6* in the digestive gland after *V. parahaemolyticus* infection are shown in Figure 5. The expression levels of *β-defensin* and *mytimacin 6* in the digestive gland of abalone were significantly increased after *V. parahaemolyticus* infection (*p* < 0.05). The mRNA level of *β-defensin* was significantly increased in AA0, AA50, AA100 and AA200 groups at 12 h and 24 h, and reached their apex at 12 h in AA200, AA500, AA1000 and AA5000 groups with 4.51-fold, 4.74-fold, 5.20-fold and 5.47-fold, respectively. The fold change of *mytimacin 6* in the digestive gland of abalone was significantly upregulated from 12 h to 24 h in the AA0, AA50, AA100, AA200 and AA500 groups, and from 6 h to 72 h in the AA1000 and AA5000 groups. The highest expression level of mytimacin 6 was observed at 12 h in the AA5000 group with 5.82-fold.

### 3.7. Expressions of Anti-Oxidation-Related and Apoptosis-Related Genes and Proteins in Digestive Gland and Gill

**Figure 6:**
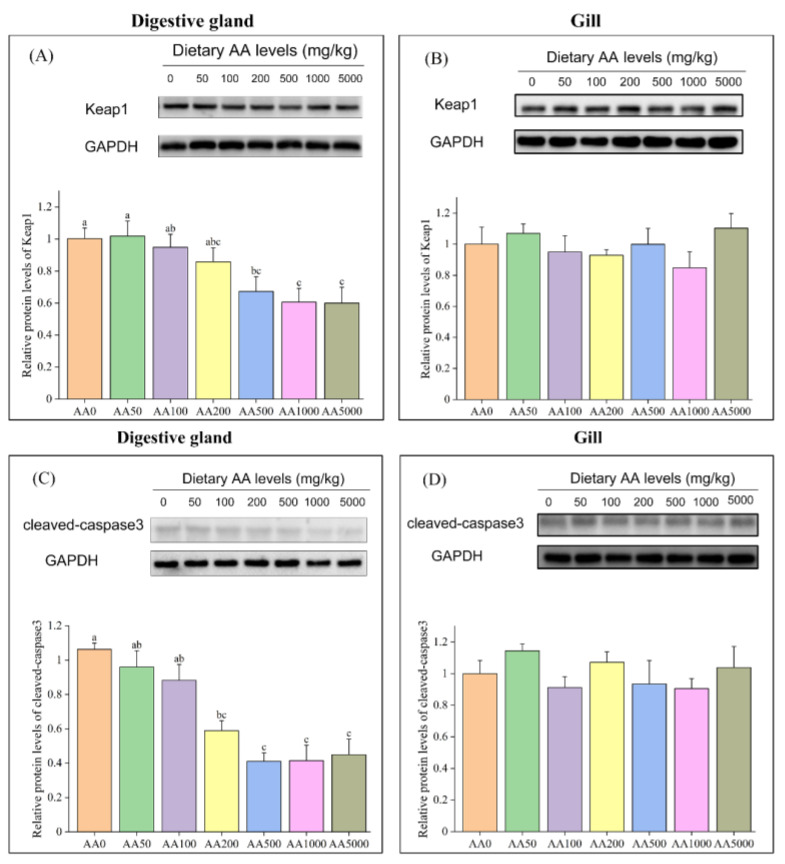
Protein expressions of Keap1 and cleaved-caspase3 in the digestive gland and gill of abalone (*Haliotis discus hannai*) after a 100-day feeding trial. (**A**) Protein expressions of Keap1 in the digestive gland of abalone. (**B**) Protein expressions of Keap1 in abalone gill. (**C**) Protein expressions of cleaved-caspase3 in the digestive gland of abalone. (**D**) Protein expressions of cleaved-caspase3 in abalone gill. All data expressed as mean ± SE (*n* = 3). The different lowercase letters indicate significant differences (*p* < 0.05).

The mRNA levels of *nf-e2-related factor-2* (*nrf2*), *gpx*, *cat* and *cuznsod* in the digestive gland of abalone were significantly elevated with graded levels of dietary AA (*p* < 0.05) (Table 11). In contrast, the mRNA and protein levels of Keap1 were significantly reduced after dietary AA (*p* < 0.05) and the *glutathione-s-transferase* (*gst*) was not affected by AA (Figure 6, Table 11) (*p* > 0.05). In the gill, the mRNA levels of *nrf2* and *gpx* were significantly increased by dietary AA (*p* < 0.05) (Table 11). However, the expressions of *keap1*, *cat*, *gst* and *cuznsod* in the gill were not subject to graded levels of dietary AA (*p* > 0.05). No significant effect of dietary AA on the protein expression of Keap1 in the gill of abalone was observed (*p* > 0.05) (Figure 6).

What stands out in Table 12 is that the expression levels of *bax* and *caspase3* in the digestive gland of abalone were significantly decreased after graded levels of dietary AA (*p* < 0.05). Meanwhile, the mRNA level of *bcl-2* was significantly upregulated (*p* < 0.05). However, the expression of *caspase7* was not affected by dietary AA (*p* > 0.05). The cleaved-caspase3 protein in the digestive gland was significantly reduced after supplementation of AA (*p* < 0.05) (Figure 6). In the gill, the mRNA levels of *caspase7* and *bcl-2* were significantly decreased and increased, respectively, after dietary AA (*p* < 0.05) (Table 12). Nevertheless, the expressions of *bax* and *caspase3* were not significantly different among all the treatments (*p* > 0.05). In addition, the protein expression of cleaved-caspase3 in the gill of abalone was not affected by graded levels of dietary AA (*p* > 0.05) (Figure 6).

## 4. Discussion

### 4.1. Ascorbic Acid Content in Different Tissues

In the present study, the AA contents in the muscle, gill and digestive gland increased with increasing dietary AA levels. A similar result was also found in Vundu (*Heterobranchus longifilis*), Japanese eel and Chu’s croaker (*Nibea coibor*) [27,56,57]. The AA content was highest in the gill, followed by the digestive gland of the abalone. However, the AA contents in the gill, digestive gland and muscle of abalone did not reach their saturation after 4821.17 mg/kg of dietary AA. In the Chinese sucker (*Myxocyprinus asiaticus*), the AA content in the liver was significantly increased by dietary AA [58]. The increased AA content in the liver of the Chinese sucker after dietary AA was correlated with liver health. In grass carp, the AA contents in the head kidney, spleen and skin were significantly elevated with the increasing dietary AA supplementation [35]. The head kidney, spleen and skin are the important immune organs in aquatic animals [59,60,61]. These results demonstrate that the increased AA content in the digestive gland and gill of abalone were closely related to the health of abalone after dietary AA and that AA accumulation clearly reflects the levels of dietary AA exposure.

### 4.2. Role of Ascorbic Acid in the Regulation of Immunity of Abalone

Hemocytes, as versatile cellular components of hemolymph in mollusks, are responsible for many aspects of molluscan life, such as immune response, biomineralization, cell-cell communication and regeneration [62,63]. Therefore, THC, RB and PA of hemolymph can directly reflect the immunity of the organism. AA was closely associated with THC, RB and PA of hemocytes, resulting in activation of the adaptive immune system [64,65].

In the present study, the THC, RB and PA in the hemolymph of abalone were significantly increased by dietary AA. It is indicated that graded levels of dietary AA succeeded in enhancing the immune function in abalone. Similar results were observed in Nile tilapia and grass shrimp (*Penaeus monodon*) [64,66]. The RB in serum of red swamp crayfish (*Procambarus clarkii*) and PA in serum of hybrid sorubim catfish (*Pseudoplatystoma reticulatum* × *P. corruscans*) were significantly decreased after a high level of dietary AA supplementation (321.38 mg/kg and 850 mg/kg, respectively) [67,68]. However, no significant decline in THC, RB and PA in the hemolymph of abalone was observed in the treatment with high levels of dietary AA supplementation (4821.17 mg/kg).

Lysozyme, C3 and C4 play pivotal roles in innate immunity and protect animals from pathological infection [69,70,71]. In the present study, the LZM in the CFH of abalone was significantly increased after graded levels of dietary AA. This was similar to the results of previous studies in cobia (*Rachycentron canadum*), Korean rockfish (*Sebastes schlegelii*) and grouper (*Epinephelus malabaricus*) [29,72,73]. AA deficiency decreased C3 and C4 contents in grass carp, and dietary AA could significantly enhance immune parameters such as C3 and C4 in largemouth bass [35,74]. In the present study, however, the C4 content in the CFH of abalone was not influenced by dietary AA levels. There was an increasing trend of C3 content in the CFH of abalone, although the C3 content was not significantly improved by dietary AA. These results revealed that dietary supplementation of AA could enhance the ability to eliminate infection of pathogens by LZM and C3, not C4, to improve immune barriers of abalone.

Innate immunity is vital for abalone because of the absence of adaptive immunity in invertebrates [43,49]. TLRs play vital roles in the innate immune system against various microbes [75]. PAMP-activated TLRs recruit specific adaptor molecules to activate transcription factors, such as NF-κB and AP-1, which determine the outcome of the innate immune response [76]. TLR signaling is classified into two distinct pathways: MyD88-dependent and MyD88-independent, which induce various cytokines such as TNF-α and IL-1β in mammals and other aquatic animals [77,78]. In the MyD88-dependent pathway, MyD88 interacts with IRAK4 and then interacts with TRAF6 [79]. TRAF6 can activate TGF-β-activated kinase 1 (TAK1) and TAK1-binding proteins (TABs) [80]. The TAK1 activates the IKK complex, which can phosphorylate IκBα that binds to NF-κB subunits (consist of p50 and p65) and phosphorylates MAPKs and JNK, which promote the translocation of AP-1 into the nucleus [81]. The destruction of IκB facilitates the nuclear translocation of NF-κB [82]. The nuclear NF-κB and AP-1 target the transcription of cytokines, such as TNF-α and IL-1β, which are well recognized as pro-inflammatory mediators that promoted inflammation [76,77,83]. In the MyD88-independent pathway, the TRAM is required and occupies a pivotal role in this pathway [84]. The intracellular components downstream of the TLR signaling pathway are commonly and highly conserved between vertebrates and invertebrates [60,85]. The intrahepatic expression of TLR4 was downregulated in AA-administrated mice (*Mus musculus*) [86]. AA exerted beneficial hepatoprotection against concanavalin A-induced immunological hepatic injury in mice by inhibition of NF-κB signal pathway [87,88]. Thus, it could be inferred from these results that AA can affect the TLR signaling pathway. However, it remains unknown how AA regulates the TLR signaling pathway in aquatic animals, especially in mollusks.

**Figure 7:**
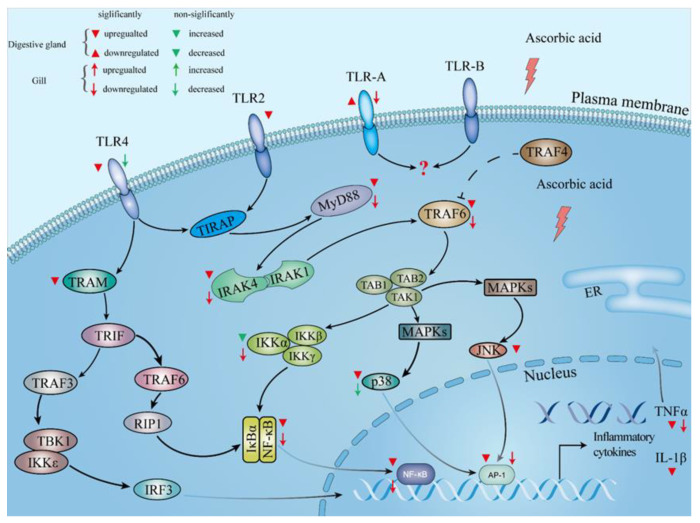
The potential TLR signaling pathway in the digestive gland and gill of abalone (*Haliotis discus hannai*) after a 100-day feeding trial. The upregulated and downregulated genes are marked by flammulated up-triangle (digestive gland) or up-arrow (gill), and down-triangle (digestive gland) or down-arrow (gill), respectively. The green up-triangle (digestive gland) or up-arrow (gill) and down-triangle (digestive gland) or down-arrow (gill) indicate the increasing and decreasing trend of the genes, but their expression levels were not significant. The signaling pathway of TLR-A and TLR-B is unclear in the recent study and marked by “?”.

In the present study, the mRNA levels of *tlr2* and *tlr4* in the digestive gland were significantly downregulated by graded levels of dietary AA. It is suggested that the TLR signaling pathway was triggered by AA in the digestive gland of abalone (Figure 7). The *tlr-a* and *tlr-b* had broader pattern recognition capacity and were involved in antibacterial and antiviral immunity of abalone [89]; however, the underlying mechanism of *tlr-a*/*b* is still unknown. However, the level of *tlr-b* was not affected by dietary AA, and *tlr-a* was significantly upregulated by AA in the present study. Further investigations are needed. The expressions of *myd88*, *irak4* and *traf6*, and the protein expression of MyD88, in the digestive gland of abalone were downregulated by supplementation of AA, and the level of *traf4* was not influenced by dietary AA. The TRAF4 acts as a silencer in TLR-mediated signaling by suppressing TRAF6 [90]. It is indicated that the inhibition of the TLR pathway by dietary AA was not mediated through the *traf4* in the digestive gland of abalone. The mRNA level of *iκbα* in the digestive gland was not influenced by AA. However, the NF-κB can be transferred to the nucleus only after IκBα phosphate degradation [91]. The mRNA expressions of *nf-κb* and protein expression of nuclear NF-κB p65 in the digestive gland were significantly downregulated by dietary AA. These results suggested that AA might suppress the phosphorylation of IκBα protein to reduce the nuclear NF-κB. IKKβ can activate NF-κB p65 to trigger the NF-κB canonical pathway, and IKKα can activate NF-κB p52 to trigger the NF-κB non-canonical pathway [92]. It is indicated that dietary AA could activate the NF-κB canonical pathway in the digestive gland of abalone. Furthermore, the mRNA expressions of *tram*, *p38 mapk*, *jnk*, *ap-1* and *tnf-α*, and the protein level of mature IL-1β were significantly reduced by dietary AA. These results suggest that dietary AA could inhibit the TLR signaling pathway through the Myd88-dependent pathway via NF-κB, p38 mapk/ap-1 and JNK/ap-1, and Myd88-independent pathways to alleviate inflammation in the digestive gland of abalone. In the gill, AA also affected the TLR signaling pathway (Figure 7). However, the mRNA levels of *tram*, *p38 mapk* and *jnk*, and protein expression of mature IL-1β were not significantly different. These results indicated that dietary AA could suppress the TLR signaling pathway through the Myd88-dependent pathway, not the MyD88-independent. The inhibited TLR pathway alleviated inflammation in the gill of abalone via NF-κB and p38 mapk/ap-1 pathway, not the JNK/ap-1 pathway. Dietary AA had a stronger effect on the TLR pathway in the digestive gland than that in the gill of abalone.

Antimicrobial peptides (AMPs) from fish and invertebrates exhibit broad-spectrum antimicrobial activity in vitro and in vivo [93,94]. Arginase can be reduced by AP-1 and plays an essential role in the anti-inflammatory process [95,96]. In the present study, the mRNA levels of *β-defensin* and *arginase-I* were significantly increased, and dietary AA did not influence the *mytimacin 6* level in the digestive gland. In contrast, the mRNA expression of *mytimacin 6* was significantly increased, and the *β-defensin* and *arginase-I* levels in the gill were not affected by dietary AA. However, the *β-defensin* mRNA level in the gill of grass carp was upregulated with dietary AA levels [36]. Thus, it is illustrated that the role of dietary AA in the regulation of immunity was species-specific. These results suggest that dietary AA could induce expression levels of AMPs to enhance the innate immunity of abalone with distinct strategies in the gill and digestive gland. The enhancement of innate immunity by dietary AA in the digestive gland was superior to that in the gill.

*Vibrio parahaemolyticus*, a kind of gram-negative bacteria, was reported to cause outbreaks of vibriosis in farmed abalone [41,42]. The expressions of immune-related genes in the digestive gland were analyzed after *V. parahaemolyticus* infection. In the present study, the mRNA expressions of *tlr4*, *myd88*, *tram*, *nf-κb*, *ap-1* and *tnf-α* were significantly upregulated, illustrating that the MyD88-dependent and MyD88-independent pathways of TLR signaling in abalone were triggered by *V. parahaemolyticus*. The highest fold change of these genes was decreased, and the immune response of abalone was more prompt with the increase of AA supplementation. These results suggest that graded levels of dietary AA could reduce inflammation in abalone. In addition, the mRNA levels of *β-defensin* and *mytimacin 6* were significantly upregulated after *V. parahaemolyticus* infection. The highest fold changes of *β-defensin* and *mytimacin 6* were increased, and the duration of the significantly increased fold change of *β-defensin* and *mytimacin 6* was prolonged with the increase of dietary AA. These results suggest that dietary AA could improve the resistance of abalone to pathogens. AA can alleviate inflammation induced by chlorpyrifos in Nile tilapia [66]. Xu et al. reported that the mRNA levels of *tnf-α* and *nf-κb* in grass carp were downregulated with increased dietary AA after *Aeromonas hydrophila* infection, and the SR was increased after infection [35]. In zebrafish, the molecular hydrogen increased the SR after *A. hydrophila* infection, and the pro-inflammatory immune response genes, such as NF-κB, were also downregulated [97]. These results suggest that a decrease in the expression of pro-inflammatory cytokines could improve the SR for aquatic animals. The present study demonstrated that dietary AA could alleviate inflammation and increase the survival of abalone after *V. parahaemolyticus* infection in a dose-dependent manner. These results suggested that dietary AA could improve the innate immunity of abalone against pathogenic microbes and promote stress resistance capacity.

### 4.3. Role of Ascorbic Acid in the Regulation of Anti-oxidative Capacity of Abalone

**Figure 8:**
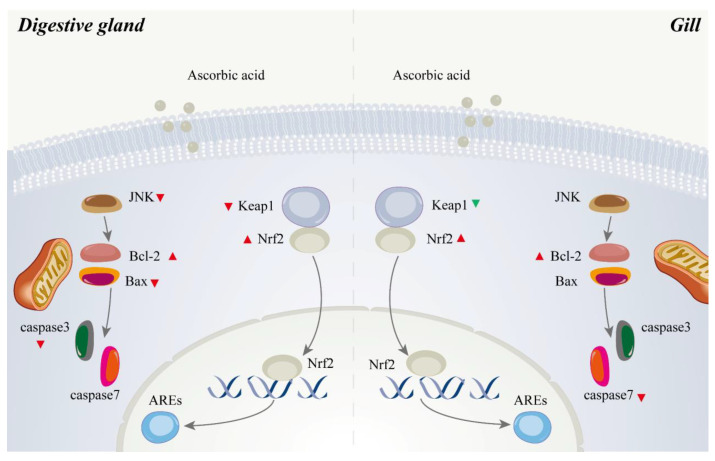
The potential pathway of apoptosis and anti-oxidation in the digestive gland and gill of abalone (*Haliotis discus hannai*) after a 100-day feeding trial. The upregulated and downregulated genes are marked by flammulated up-triangle and down-triangle, respectively. The green up-triangle and green down-triangle indicate the increasing and decreasing trend of the genes, but their expression levels were not significant.

AA serves as an anti-oxidant and prevents other compounds from being oxidized [98]. The SOD, CAT and GPX activities and T-AOC and content of GSH were involved in the anti-oxidative defense mechanism [99]. The SOD, CAT and GPX activities and T-AOC and content of GSH were significantly upregulated in the present study. The MDA content in the CFH of abalone was significantly reduced by dietary AA. Similar results were also observed in the serum of largemouth bass and yellow catfish (*Pelteobagrus fulvidraco*) [100,101]. However, in largemouth bass, SOD activity in muscle showed an opposite pattern of change to that in the liver after dietary AA [100]. It is suggested that there are differences in the function of AA upon accumulation in different tissues. These results suggested that supplementation of AA in the diet could improve the anti-oxidative capacity of abalone. Under normal conditions, Nrf2 is inhibited by Keap1 [102]. The Nrf2 can activate the transcription of anti-oxidative response element (AREs) genes. The anti-oxidative gene expressions and protein levels revealed that dietary AA could trigger the Keap1-Nrf2-AREs pathway to enhance the anti-oxidative capacity of abalone (Figure 8).

### 4.4. Role of Ascorbic Acid in the Regulation of Apoptosis of Abalone

The activation of the JNK signaling cascade can reduce the expression of Bcl-2 and increase the level of Bax, thereby causing apoptosis [103,104]. Bcl-2, an anti-apoptotic protein, can reduce the levels of caspase-3 and caspase-7, which are executioner caspases and share a common role in apoptosis [105]. In contrast, the Bax can activate caspase-3 and caspase-7 [106]. In the present study, mRNA levels of *bax* and *caspase3* and the protein expression of cleaved-caspase3 in the digestive gland were significantly decreased after dietary AA. The mRNA level of *Bcl-2* was significantly upregulated by dietary AA. In the gill, mRNA levels of *caspase7* and *bcl-2* were significantly decreased and increased, respectively, by dietary AA. Dietary AA supplementation can attenuate low temperature-induced cell apoptosis in pufferfish (*Takifugu obscurus*) [107]. Similar results were also observed in grass carp and mussels (*Mytilus galloprovincialis*) [35,36,108]. The present study indicated that the JNK-Bcl-2/Bax pathway in abalone was suppressed by dietary AA to enhance its anti-apoptosis capacity (Figure 8).

## 5. Conclusions

In summary, the TLR-MyD88-dependent and TLR-MyD88-independent signaling pathways in the digestive gland of abalone were suppressed by 919.99 mg/kg and 4821.17 mg/kg of dietary AA. Meanwhile, only the TLR-MyD88-dependent pathway in the gill of abalone was depressed by 919.99 mg/kg and 4821.17 mg/kg of dietary AA to reduce the inflammation in abalone. The supplementation of 919.99 mg/kg and 4821.17 mg/kg of dietary AA could enhance the anti-oxidative capacity by triggering the Keap1-Nrf2-AREs pathway. They could improve the anti-apoptosis ability via the JNK-Bcl-2/Bax signaling cascade. Supplementation of 919.99 mg/kg of dietary AA was adequate for abalone with excellent immunity, anti-oxidative capacity and anti-apoptosis ability. These findings provided the theoretical basis and reference data for the diet formulation and health regulation of abalone.

## Figures and Tables

**Table 1:** Formulation and proximate compositions of the basal diet.

Ingredient	Content (g/100 g, Dry Matter)
Casein (vitamin-free) ^a^	25.00
Gelatin ^b^	6.00
SO/MFO (1:1) ^c^	3.50
Dextrin ^b^	33.50
Vitamin mix ^d^	2.00
Mineral mix ^e^	4.50
Sodium alginate ^b^	20.00
Choline chloride ^b^	0.50
Carboxymethyl cellulose ^b^	5.00
Proximate analysis (dry matter)	
Crude protein (%)	31.25
Crude lipid (%)	3.42
Ash (%)	10.67

^a^ Sigma Chemical, St Louis, MO, USA. ^b^ Shanghai Chemical, Shanghai, China. ^c^ Soybean oil (SO) and Menhaden fish oil (MFO). ^d^ Vitamin mix (ascorbic acid free), each 1000 g of diet contained: thiamin HCL, 120 mg; riboflavin, 100 mg; folic acid, 30 mg; pyridoxine HCl, 40 mg; niacin, 800 mg; Ca pantothenate, 200 mg; inositol, 4000 mg; biotin, 12 mg; vitamin B12, 0.18 mg; vitamin E, 450 mg; menadione, 80 mg; retinol acetate, 100,000 IU; vitamin D_3_, 2000 IU. ^e^ Mineral mix, each 1000 g of diet contained: NaCl, 0.4 g; MgSO_4_·7H_2_O, 6.0 g; NaH_2_PO_4_·2H_2_O, 10.0 g; KH_2_PO_4_, 20.0 g; Ca(H_2_PO_4_)_2_·H_2_O, 8.0 g; Fe-citrate, 1.0 g; ZnSO_4_·7H_2_O, 141.2 mg; MnSO_4_·H_2_O, 64.8 mg; CoCl_2_·6H_2_O, 0.4 mg; KIO_3_, 1.2 mg; CuSO_4_·5H_2_O, 12.4 mg; Na_2_SeO_3_·5H_2_O, 0.4 mg.

**Table 2:** Effects of dietary ascorbic acid on survival and growth performance of abalone (*Haliotis discus hannai*) after a 100-day feeding trial.

Group	Initial Weight (g)	Initial Shell Length (mm)	WGR (%)	DISL (μm/Day)	SR (%)
AA0	12.02 ± 0.003	48.40 ± 0.53	30.38 ± 0.11	18.23 ± 3.05	84.44 ± 2.22
AA50	12.01 ± 0.004	48.40 ± 0.07	31.11 ± 0.33	18.67 ± 0.96	85.19 ± 0.74
AA100	12.01 ± 0.003	48.52 ± 0.3	31.52 ± 2.06	18.81 ± 2.07	82.96 ± 5.19
AA200	12.01 ± 0.002	48.66 ± 0.24	30.06 ± 0.87	19.48 ± 0.24	80.74 ± 0.74
AA500	12.02 ± 0.007	48.43 ± 0.23	30.27 ± 1.43	17.92 ± 1.61	88.15 ± 1.48
AA1000	12.01 ± 0.001	48.19 ± 0.21	29.32 ± 1.73	20.01 ± 2.38	88.15 ± 1.96
AA5000	12.01 ± 0.003	48.49 ± 0.33	29.48 ± 1.4	19.01 ± 2.68	82.96 ± 2.67

All data expressed as mean ± SE (*n* = 3). SR: survival rate; WGR: weight gain rate; DISL: daily increment in shell length.

**Table 3:** Effects of dietary ascorbic acid on total hemocyte counts (THC), respiratory burst (RB) and phagocytic activity (PA) in abalone (*Haliotis discus hannai*) hemolymph after a 100-day feeding trial.

Group	THC (×10^7^ Cells/mL)	RB (OD_630_/10^7^ Cells·mL^−1^)	PA (%)
AA0	1.07 ± 0.05 ^d^	0.17 ± 0.012 ^c^	27.11 ± 2.91 ^c^
AA50	1.09 ± 0.06 ^d^	0.18 ± 0.009 ^c^	29.96 ± 1.45 ^c^
AA100	1.11 ± 0.05 ^d^	0.17 ± 0.007 ^c^	30.92 ± 3.76 ^c^
AA200	1.23 ± 0.02 ^cd^	0.20 ± 0.012 ^c^	35.87 ± 1.62 ^bc^
AA500	1.37 ± 0.01 ^bc^	0.27 ± 0.009 ^b^	38.43 ± 2.06 ^abc^
AA1000	1.65 ± 0.06 ^a^	0.35 ± 0.013 ^a^	46.42 ± 2.73 ^ab^
AA5000	1.60 ± 0.08 ^ab^	0.32 ± 0.010 ^a^	48.92 ± 2.65 ^a^

All data expressed as mean ± SE (*n* = 3). The different lowercase letters behind the number indicate significant differences (*p* < 0.05).

**Table 4:** Effects of dietary ascorbic acid on the lysozyme activity, C3 and C4 content in cell-free hemolymph of abalone (*Haliotis discus hannai*) after a 100-day feeding trial.

Group	LZM (U/mL)	C3 (mg/L)	C4 (mg/L)
AA0	22.92 ± 2.08 ^c^	27.56 ± 2.77	51.720 ± 3.390
AA50	21.67 ± 1.82 ^c^	35.01 ± 3.40	54.960 ± 0.950
AA100	20.83 ± 2.08 ^c^	38.79 ± 3.29	54.070 ± 3.600
AA200	33.33 ± 2.08 ^bc^	41.77 ± 7.63	56.390 ± 0.480
AA500	37.50 ± 3.61 ^b^	41.65 ± 3.31	53.660 ± 4.960
AA1000	56.25 ± 3.61 ^a^	48.46 ± 5.93	56.420 ± 2.540
AA5000	52.08 ± 4.17 ^a^	41.82 ± 8.68	57.840 ± 1.270

All data were expressed as Mean ± SE (*n* = 3). The different lowercase letters in the column indicate significant differences (*p* < 0.05). LZM: lysozyme; C3: complement component 3; C4: complement component 4.

**Table 5:** Effects of dietary ascorbic acid on the anti-oxidative capacity in the cell-free hemolymph of abalone (*Haliotis discus hannai*) after a 100-day feeding trial.

Group	T-AOC (μmol/mL)	SOD (U/mL)	CAT (U/mL)	GSH (μmol/L)	GPX (U/mL)	MDA (nmol/mL)
AA0	0.34 ± 0.01 ^c^	8.89 ± 0.06 ^c^	1.51 ± 0.20 ^c^	3.86 ± 0.15 ^d^	2.14 ± 0.11 ^c^	3.70 ± 0.33 ^a^
AA50	0.33 ± 0.00 ^c^	8.83 ± 0.12 ^c^	1.37 ± 0.15 ^c^	4.57 ± 0.22 ^cd^	1.98 ± 0.07 ^c^	3.80 ± 0.37 ^a^
AA100	0.34 ± 0.02 ^c^	10.64 ± 1.02 ^bc^	1.75 ± 0.41 ^bc^	5.28 ± 0.19 ^cd^	2.02 ± 0.08 ^c^	3.84 ± 0.20 ^a^
AA200	0.34 ± 0.01 ^bc^	11.12 ± 0.89 ^abc^	2.68 ± 0.10 ^ab^	5.66 ± 0.40 ^c^	2.39 ± 0.11 ^bc^	3.70 ± 0.20 ^a^
AA500	0.39 ± 0.02 ^abc^	12.36 ± 0.29 ^ab^	2.80 ± 0.20 ^ab^	7.80 ± 0.19 ^b^	3.09 ± 0.29 ^b^	3.38 ± 0.09 ^ab^
AA1000	0.41 ± 0.01 ^a^	13.38 ± 0.44 ^ab^	3.68 ± 0.23 ^a^	10.27 ± 0.59 ^a^	4.28 ± 0.27 ^a^	2.36 ± 0.08 ^b^
AA5000	0.40 ± 0.01 ^ab^	13.54 ± 0.44 ^a^	3.74 ± 0.20 ^a^	9.60 ± 0.54 ^a^	4.24 ± 0.11 ^a^	2.41 ± 0.28 ^b^

All data expressed as mean ± SE (*n* = 3). The different lowercase letters in the column indicate significant differences (*p* < 0.05). T-AOC: total anti-oxidative capacity; SOD: superoxide dismutase; CAT: catalase; GSH: reduced glutathione; MDA: malondialdehyde; GPX: glutathione peroxidase.

**Table 6:** Expression levels of *tlr2*, *tlr4*, *tlr-a*, *tlr-b*, *myd88*, *tram*, *irak4*, *traf4* and *traf6* in the digestive gland of abalone (*Haliotis discus hannai*) after a 100-day feeding trial.

Gene	AA0	AA50	AA100	AA200	AA500	AA1000	AA5000
*tlr2*	1.29 ± 0.08 ^a^	0.91 ± 0.03 ^ab^	0.78 ± 0.06 ^abc^	0.70 ± 0.10 ^bc^	0.66 ± 0.10 ^bc^	0.53 ± 0.09 ^c^	0.60 ± 0.02 ^bc^
*tlr4*	0.90 ± 0.09 ^a^	0.71 ± 0.08 ^ab^	0.71 ± 0.06 ^ab^	0.69 ± 0.06 ^b^	0.64 ± 0.03 ^bc^	0.47 ± 0.03 ^cd^	0.43 ± 0.06 ^d^
*tlr-a*	1.22 ± 0.09 ^b^	1.57 ± 0.24 ^ab^	1.70 ± 0.29 ^ab^	1.77 ± 0.10 ^ab^	2.05 ± 0.34 ^a^	2.18 ± 0.17 ^a^	2.20 ± 0.19 ^a^
*tlr-b*	1.07 ± 0.12	1.03 ± 0.09	0.98 ± 0.12	0.91 ± 0.08	0.93 ± 0.10	0.94 ± 0.05	1.04 ± 0.07
*myd88*	0.99 ± 0.08 ^a^	0.98 ± 0.06 ^a^	0.93 ± 0.04 ^a^	0.90 ± 0.09 ^a^	0.67 ± 0.08 ^b^	0.47 ± 0.06 ^bc^	0.43 ± 0.04 ^c^
*tram*	1.03 ± 0.10 ^a^	1.00 ± 0.15 ^ab^	0.92 ± 0.10 ^ab^	0.95 ± 0.12 ^ab^	0.82 ± 0.09 ^ab^	0.65 ± 0.11 ^b^	0.78 ± 0.05 ^ab^
*irak4*	1.11 ± 0.10 ^a^	0.90 ± 0.09 ^ab^	0.88 ± 0.09 ^ab^	0.88 ± 0.10 ^ab^	0.67 ± 0.06 ^bc^	0.65 ± 0.06 ^bc^	0.61 ± 0.06 ^c^
*traf6*	0.97 ± 0.03 ^ab^	0.98 ± 0.08 ^a^	0.71 ± 0.07 ^bc^	0.69 ± 0.06 ^c^	0.61 ± 0.03 ^c^	0.52 ± 0.05 ^c^	0.60 ± 0.04 ^c^
*traf4*	1.00 ± 0.10	0.97 ± 0.07	1.01 ± 0.07	1.16 ± 0.13	0.96 ± 0.07	0.96 ± 0.11	0.94 ± 0.10

All data are expressed as mean ± SE (*n* = 3). The different lowercase letters in each row indicate significant differences (*p* < 0.05). *tlr2: toll-like receptor 2; tlr4: toll-like receptor 4; tlr-a: toll-like receptor a; tlr-b: toll-like receptor b; myd88: myeloid differentiation primary response gene 88; tram: trif-related adaptor molecule; irak4: interleukin 1 receptor-associated kinase 4; traf6: tumor necrosis factor receptor-associated factor 6; traf4: tumor necrosis factor (tnf) receptor-associated factor 4.*

**Table 7:** Expression levels of *ikkα*, *iκbα*, *p38 mapk*, *jnk*, *nf-κb*, *ap-1*, *tnf-α* and *il 16* in the digestive gland of abalone (*Haliotis discus hannai*) after a 100-day feeding trial.

Gene	AA0	AA50	AA100	AA200	AA500	AA1000	AA5000
*ikk* *α*	0.88 ± 0.08	0.83 ± 0.05	0.82 ± 0.08	0.84 ± 0.05	0.70 ± 0.06	0.70 ± 0.08	0.73 ± 0.06
*i* *κbα*	1.13 ± 0.06	1.07 ± 0.08	1.20 ± 0.07	1.00 ± 0.09	1.06 ± 0.04	1.14 ± 0.06	0.97 ± 0.07
*p38 mapk*	1.05 ± 0.13 ^a^	0.95 ± 0.07 ^ab^	0.93 ± 0.01 ^ab^	0.92 ± 0.09 ^ab^	0.75 ± 0.08 ^bc^	0.54 ± 0.05 ^c^	0.57 ± 0.02 ^c^
*jnk*	0.98 ± 0.04 ^a^	0.88 ± 0.06 ^ab^	0.92 ± 0.05 ^a^	0.81 ± 0.08 ^ab^	0.70 ± 0.06 ^bc^	0.48 ± 0.04 ^d^	0.59 ± 0.06 ^cd^
*nf-κb*	0.91 ± 0.07 ^a^	0.91 ± 0.05 ^a^	0.92 ± 0.03 ^a^	0.73 ± 0.05 ^ab^	0.70 ± 0.04 ^b^	0.58 ± 0.05 ^b^	0.70 ± 0.11 ^b^
*ap-1*	1.25 ± 0.08 ^a^	0.95 ± 0.06 ^b^	0.93 ± 0.02 ^b^	0.89 ± 0.07 ^b^	0.91 ± 0.06 ^b^	0.70 ± 0.05 ^c^	0.74 ± 0.07 ^b^
*tnf-α*	1.07 ± 0.06 ^a^	0.88 ± 0.03 ^ab^	0.76 ± 0.12 ^ab^	0.81 ± 0.16 ^ab^	0.66 ± 0.11 ^b^	0.60 ± 0.07 ^b^	0.65 ± 0.07 ^b^
*il 16*	0.82 ± 0.02	0.88 ± 0.04	0.81 ± 0.04	0.84 ± 0.06	0.78 ± 0.08	0.86 ± 0.10	0.84 ± 0.04

All data expressed as mean ± SE (*n* = 3). The different lowercase letters in each row indicate significant differences (*p* < 0.05). *ikkα: iκb kinase α. iκbα: nf-κb inhibitor α. p38 mapk: p38 mitogen-activated protein kinase. jnk: c-jun n-terminal kinase. nf-κb: nuclear factor-κb. ap-1: activator protein 1. tnf-α: tumor necrosis factor α. il 16: interleukin 16.*

**Table 8:** Expression levels of *tlr2*, *tlr4*, *tlr-a*, *tlr-b*, *myd88*, *tram*, *irak4*, *traf4* and *traf6* in the gill of abalone (*Haliotis discus hannai*) after a 100-day feeding trial.

Gene	AA0	AA50	AA100	AA200	AA500	AA1000	AA5000
*tlr2*	0.91 ± 0.09	0.83 ± 0.08	0.99 ± 0.06	0.89 ± 0.05	0.88 ± 0.07	0.96 ± 0.11	0.93 ± 0.10
*tlr4*	1.04 ± 0.06	0.92 ± 0.09	1.05 ± 0.10	0.87 ± 0.09	0.75 ± 0.08	0.76 ± 0.07	0.74 ± 0.05
*tlr-a*	0.75 ± 0.06 ^a^	0.66 ± 0.04 ^ab^	0.52 ± 0.05 ^c^	0.54 ± 0.02 ^bc^	0.42 ± 0.02 ^cd^	0.38 ± 0.03 ^d^	0.38 ± 0.03 ^d^
*tlr-b*	1.10 ± 0.08	1.02 ± 0.08	1.18 ± 0.06	1.19 ± 0.10	1.08 ± 0.08	1.14 ± 0.08	1.06 ± 0.10
*myd88*	0.92 ± 0.09 ^a^	0.91 ± 0.06 ^ab^	0.90 ± 0.09 ^ab^	0.85 ± 0.10 ^ab^	0.63 ± 0.10 ^ab^	0.57 ± 0.12 ^b^	0.62 ± 0.09 ^ab^
*tram*	1.09 ± 0.08	1.00 ± 0.06	0.99 ± 0.04	1.00 ± 0.07	1.03 ± 0.07	0.99 ± 0.06	1.09 ± 0.06
*irak4*	0.97 ± 0.04 ^a^	0.86 ± 0.08 ^ab^	0.85 ± 0.04 ^ab^	0.78 ± 0.03 ^abc^	0.64 ± 0.09 ^c^	0.64 ± 0.05 ^c^	0.75 ± 0.02 ^bc^
*traf6*	1.06 ± 0.05 ^a^	0.92 ± 0.08 ^a^	0.94 ± 0.06 ^a^	0.94 ± 0.08 ^a^	0.84 ± 0.13 ^ab^	0.65 ± 0.06 ^b^	0.67 ± 0.06 ^b^
*traf4*	1.33 ± 0.09	1.25 ± 0.13	1.26 ± 0.09	1.32 ± 0.09	1.37 ± 0.09	1.32 ± 0.10	1.31 ± 0.12

All data expressed as mean ± SE (n = 3). Lowercase letters in each row indicate significant differences (*p* < 0.05). *tlr2: toll-like receptor 2; tlr4: toll-like receptor 4; tlr-a: toll-like receptor a; tlr-b: toll-like receptor b; myd88: myeloid differentiation primary response gene 88; tram: trif-related adaptor molecule; irak4: interleukin 1 receptor-associated kinase 4; traf6: tumor necrosis factor receptor-associated factor 6; traf4: tumor necrosis factor (tnf) receptor-associated factor 4*.

**Table 9:** Expression levels of *ikkα*, *iκbα*, *p38 mapk*, *jnk*, *nf-κb*, *ap-1*, *tnf-α* and *il 16* in the gill of abalone (*Haliotis discus hannai*) after a 100-day feeding trial.

Gene	AA0	AA50	AA100	AA200	AA500	AA1000	AA5000
*ikkα*	0.99 ± 0.08 ^a^	0.83 ± 0.08 ^ab^	0.87 ± 0.08 ^ab^	0.73 ± 0.07 ^bc^	0.68 ± 0.05 ^bcd^	0.49 ± 0.04 ^d^	0.54 ± 0.05 ^cd^
*iκbα*	1.07 ± 0.01	0.92 ± 0.04	0.97 ± 0.07	0.95 ± 0.05	1.09 ± 0.08	0.97 ± 0.09	0.99 ± 0.08
*p38 mapk*	1.01 ± 0.11	0.92 ± 0.08	0.91 ± 0.09	0.96 ± 0.04	0.79 ± 0.08	0.80 ± 0.09	0.74 ± 0.08
*jnk*	1.06 ± 0.06	0.95 ± 0.06	0.98 ± 0.05	0.87 ± 0.05	0.89 ± 0.06	0.90 ± 0.06	0.98 ± 0.08
*nf-κb*	1.08 ± 0.05 ^a^	0.97 ± 0.06 ^ab^	1.02 ± 0.07 ^ab^	0.86 ± 0.05 ^bc^	0.77 ± 0.07 ^c^	0.71 ± 0.03 ^c^	0.75 ± 0.09 ^c^
*ap-1*	0.98 ± 0.05 ^a^	0.94 ± 0.03 ^ab^	0.83 ± 0.07 ^ab^	0.80 ± 0.05 ^ab^	0.90 ± 0.03 ^ab^	0.74 ± 0.05 ^b^	0.84 ± 0.11 ^ab^
*tnf-α*	1.00 ± 0.02 ^a^	0.88 ± 0.07 ^ab^	0.92 ± 0.06 ^ab^	0.82 ± 0.07 ^abc^	0.83 ± 0.12 ^abc^	0.62 ± 0.03 ^c^	0.69 ± 0.06 ^bc^
*il 16*	1.01 ± 0.06	0.94 ± 0.14	1.07 ± 0.06	0.93 ± 0.08	1.06 ± 0.09	0.93 ± 0.08	0.90 ± 0.08

All data are expressed as mean ± SE (n = 3). The different lowercase in each row indicates significant differences (*p* < 0.05). *ikkα: iκb kinase α. iκbα: nf-κb inhibitor α. p38 mapk: p38 mitogen-activated protein kinase. jnk: c-jun n-terminal kinase. nf-κb: nuclear factor-κb. ap-1: activator protein 1. tnf-α: tumor necrosis factor α. il 16: interleukin 16*.

**Table 10:** Expression levels of *β-defensin*, *mytimacin 6* and *arginase-I* in the digestive gland and gill of abalones (*Haliotis discus hannai*) after a 100-day feeding trial.

	Gene	AA0	AA50	AA100	AA200	AA500	AA1000	AA5000
Digestive gland	*β-defensin*	1.77 ± 0.09 ^c^	2.06 ± 0.07 ^bc^	2.10 ± 0.17 ^bc^	2.02 ± 0.19 ^bc^	2.51 ± 0.22 ^b^	3.28 ± 0.44 ^a^	3.13 ± 0.10 ^a^
	*mytimacin 6*	1.09 ± 0.10	1.10 ± 0.08	1.02 ± 0.10	1.14 ± 0.10	1.12 ± 0.09	1.16 ± 0.16	1.01 ± 0.10
	*arginase-I*	1.34 ± 0.10 ^c^	1.99 ± 0.14 ^bc^	2.15 ± 0.21 ^b^	2.02 ± 0.18 ^b^	3.25 ± 0.25 ^a^	2.67 ± 0.25 ^ab^	2.60 ± 0.26 ^ab^
Gill	*β-defensin*	2.45 ± 0.11	2.37 ± 0.25	2.94 ± 0.22	2.92 ± 0.19	3.01 ± 0.12	2.97 ± 0.41	3.00 ± 0.29
	*mytimacin 6*	1.71 ± 0.13 ^c^	1.94 ± 0.15 ^c^	1.88 ± 0.19 c	2.08 ± 0.12 ^bc^	2.69 ± 0.21 ^ab^	2.75 ± 0.24 ^a^	2.83 ± 0.36 ^a^
	*arginase-I*	1.98 ± 0.19	1.89 ± 0.14	1.87 ± 0.19	1.81 ± 0.16	1.85 ± 0.12	1.92 ± 0.19	1.87 ± 0.14

All data expressed as mean ± SE (*n* = 3). Different lowercase letters in each row indicate significant differences (*p* < 0.05).

**Table 11:** Expression levels of anti-oxidative-related genes in the digestive gland and gill of abalone (*Haliotis discus hannai*) after a 100-day feeding trial.

	Gene	AA0	AA50	AA100	AA200	AA500	AA1000	AA5000
Digestive gland	*nrf2*	0.92 ± 0.09 ^d^	1.11 ± 0.11 ^cd^	1.58 ± 0.17 ^bc^	1.96 ± 0.25 ^ab^	2.13 ± 0.24 ^a^	2.21 ± 0.16 ^a^	2.36 ± 0.20 ^a^
	*keap1*	1.03 ± 0.09 ^a^	1.03 ± 0.07 ^a^	1.06 ± 0.08 ^a^	0.88 ± 0.11 ^ab^	0.77 ± 0.05 ^ab^	0.59 ± 0.08 ^b^	0.62 ± 0.06 ^b^
	*gpx*	1.04 ± 0.15 ^c^	1.07 ± 0.13 ^c^	1.11 ± 0.09 ^c^	1.41 ± 0.12 ^bc^	1.64 ± 0.12 ^ab^	1.91 ± 0.18 ^a^	1.99 ± 0.20 ^a^
	*cat*	1.07 ± 0.11 ^c^	1.06 ± 0.09 ^c^	1.29 ± 0.07 ^c^	1.51 ± 0.19 ^bc^	1.89 ± 0.17 ^ab^	2.04 ± 0.20 ^a^	2.01 ± 0.23 ^ab^
	*gst*	1.26 ± 0.09	1.08 ± 0.02	1.08 ± 0.14	1.20 ± 0.06	1.07 ± 0.12	1.15 ± 0.11	1.15 ± 0.10
	*cuznsod*	1.18 ± 0.05 ^b^	1.17 ± 0.10 ^b^	1.14 ± 0.04 ^b^	1.10 ± 0.02 ^b^	1.26 ± 0.14 ^ab^	1.51 ± 0.11 ^a^	1.37 ± 0.05 ^ab^
Gill	*nrf2*	1.08 ± 0.06 ^b^	1.00 ± 0.11 ^b^	1.05 ± 0.10 ^b^	1.01 ± 0.02 ^b^	1.25 ± 0.20 ^ab^	1.54 ± 0.16 ^a^	1.54 ± 0.17 ^a^
	*keap1*	1.05 ± 0.04	1.08 ± 0.09	0.99 ± 0.08	0.98 ± 0.08	0.93 ± 0.14	0.78 ± 0.04	0.76 ± 0.06
	*gpx*	1.01 ± 0.15 ^b^	0.99 ± 0.14 ^b^	1.13 ± 0.10 ^b^	1.12 ± 0.08 ^b^	1.44 ± 0.22 ^ab^	1.85 ± 0.10 ^a^	1.89 ± 0.19 ^a^
	*cat*	1.14 ± 0.06	1.15 ± 0.17	1.15 ± 0.05	1.22 ± 0.18	1.25 ± 0.08	1.49 ± 0.08	1.33 ± 0.11
	*gst*	1.04 ± 0.15	1.04 ± 0.08	1.19 ± 0.09	1.18 ± 0.07	1.16 ± 0.05	1.40 ± 0.19	1.21 ± 0.04
	*cuznsod*	1.07 ± 0.05	1.27 ± 0.12	1.27 ± 0.06	1.24 ± 0.08	1.20 ± 0.05	1.13 ± 0.09	1.16 ± 0.08

All data are expressed as mean ± SE (n = 3). *nrf2: nf-e2-related factor-2*; *keap1: kelch-like ech-associating protein 1*; *gpx: glutathione peroxidase*; *cat*: *catalase*; *gst*: *glutathione-s-transferase*; *cuznsod*: *copper-zinc-superoxide dismutase*. The different lowercase letters in each row indicate significant differences (*p* < 0.05).

**Table 12:** Expression levels of apoptosis-related genes in the digestive gland and gill of abalone (*Haliotis discus hannai*) after a 100-day feeding trial.

	Gene	AA0	AA50	AA100	AA200	AA500	AA1000	AA5000
Digestive gland	*bax*	1.03 ± 0.06 ^a^	0.92 ± 0.12 ^ab^	0.95 ± 0.01 ^ab^	0.85 ± 0.10 ^abc^	0.68 ± 0.06 ^c^	0.66 ± 0.04 ^c^	0.74 ± 0.04 ^bc^
	*caspase3*	1.27 ± 0.06 ^a^	1.06 ± 0.08 ^ab^	1.17 ± 0.08 ^a^	1.00 ± 0.05 ^abc^	0.76 ± 0.14 ^bc^	0.64 ± 0.09 ^c^	0.65 ± 0.14 ^c^
	*caspase7*	1.23 ± 0.08	1.18 ± 0.09	1.23 ± 0.11	1.17 ± 0.09	1.18 ± 0.13	1.09 ± 0.10	1.24 ± 0.04
	*bcl-2*	0.59 ± 0.09 ^d^	0.64 ± 0.13 ^cd^	0.81 ± 0.05 ^cd^	1.17 ± 0.07 ^bc^	1.71 ± 0.16 ^ab^	1.75 ± 0.13 ^a^	1.50 ± 0.13 ^ab^
Gill	*bax*	0.95 ± 0.07	0.83 ± 0.05	0.90 ± 0.05	0.91 ± 0.05	0.94 ± 0.09	0.73 ± 0.07	0.76 ± 0.05
	*caspase3*	1.30 ± 0.06	1.32 ± 0.03	1.32 ± 0.05	1.23 ± 0.11	1.17 ± 0.09	1.08 ± 0.07	1.11 ± 0.06
	*caspase7*	1.08 ± 0.11 ^a^	0.97 ± 0.10 ^ab^	0.94 ± 0.09 ^ab^	0.69 ± 0.06 ^c^	0.74 ± 0.07 ^bc^	0.59 ± 0.05 ^c^	0.63 ± 0.05 ^c^
	*bcl-2*	1.00 ± 0.10 ^bc^	0.90 ± 0.09 ^c^	1.04 ± 0.06 ^bc^	0.99 ± 0.08 ^c^	1.06 ± 0.07 ^bc^	1.44 ± 0.13 ^ab^	1.51 ± 0.11 ^a^

All data are expressed as mean ± SE (*n* = 3). *bax: bcl2-associated x; caspase-3: apoptosis-related cysteine peptidase 3; caspase-7: apoptosis-related cysteine peptidase 7; bcl-2: apoptosis regulator bcl-2*. The different lowercase in each row indicate significant differences (*p* < 0.05).

## Data Availability

Data is contained within the article or Supplementary Material.

## Supplementary Materials

The following are available online at https://www.mdpi.com/article/10.3390/antiox10091449/s1, Supplementary Figure S1: Retention efficiency of dietary ascorbic acid in experimental diets at different intervals immersed in seawater, Supplementary Table S1: Primer used in this study for qPCR.

## References

[1] Carr A.C., Maggini S. (2017). Vitamin C and Immune Function. Nutrients.

[2] Granger M., Eck P. (2018). Chapter Seven—Dietary Vitamin C in Human Health. Adv. Food Nutr. Res..

[3] Reyes J.B.D., Kim J.H., Han G.P., Won S.Y., Kil D.Y. (2021). Effects of dietary supplementation of vitamin C on productive performance, egg quality, tibia characteristics and antioxidant status of laying hens. Livestig. Sci..

[4] Weber P., Bendich A., Schalch W. (1996). Vitamin C and human health—A review of recent data relevant to human requirements. Int. J. Vitam. Nutr. Res..

[5] Bsoul S.A., Terezhalmy G.T. (2004). Vitamin C in health and disease. J. Contemp. Dent. Pract..

[6] Parker A., Cuddihy S.L., Son T.G., Vissers M., Winterbourn C.C. (2011). Roles of superoxide and myeloperoxidase in ascorbate oxidation in stimulated neutrophils and H_2_O_2_-treated HL60 cells. Free Radic. Biol. Med..

[7] Anderson R. (1981). Ascorbate-mediated stimulation of neutrophil motility and lymphocyte transformation by inhibition of the peroxidase/H_2_O_2_/halide system in vitro and in vivo. Am. J. Clin. Nutr..

[8] Rebora A., Crovato F., Dallegri F., Patrone F. (1980). Repeated staphylococcal pyoderma in two siblings with defective neutrophil bacterial killing. Dermatology.

[9] Fisher B.J., Kraskauskas D., Martin E.J., Farkas D., Natarajan R. (2012). Mechanisms of attenuation of abdominal sepsis induced acute lung injury by ascorbic acid. Am. J. Physiol. Lung Cell Mol. Physiol..

[10] Washko P.W., Wang Y.H., Levine M. (1993). Ascorbic acid recycling in human neutrophils. J. Biol. Chem..

[11] Levy R., Shriker O., Porath A., Riesenberg K., Schlaeffer F. (1996). Vitamin C for the Treatment of Recurrent Furunculosis in Patients with Impaired Neutrophil Functions. J. Infect. Dis..

[12] Chang H.H., Chen C.S., Lin J.Y. (2009). High Dose Vitamin C Supplementation Increases the Th1/Th2 Cytokine Secretion Ratio, but Decreases *Eosinophilic Infiltration* in Bronchoalveolar Lavage Fluid of Ovalbumin-Sensitized and Challenged Mice. J. Agric. Food Chem..

[13] Oudemans-van Straaten H.M., Spoelstra-de Man A.M., de Waard M.C. (2014). Vitamin C revisited. Crit Care.

[14] Su X., Shen Z., Yang Q., Sui F., Pu J., Ma J., Ma S., Yao D., Ji M., Hou P. (2019). Vitamin C kills thyroid cancer cells through ROS-dependent inhibition of MAPK/ERK and PI3K/AKT pathways via distinct mechanisms. Theranostics.

[15] Yang M., Teng S., Ma C., Yu Y., Wang P., Yi C. (2018). Ascorbic acid inhibits senescence in mesenchymal stem cells through ROS and AKT/mTOR signaling. Cytotechnology.

[16] Amatore C., Arbault S., Ferreira D.C.M., Tapsoba I., Verchier Y. (2008). Vitamin C stimulates or attenuates reactive oxygen and nitrogen species (ROS, RNS) production depending on cell state: Quantitative amperometric measurements of oxidative bursts at PLB-985 and RAW 264.7 cells at the single cell level. J. Electroanal. Chem..

[17] Bei R. (2013). Effects of vitamin C on health: A review of evidence. Front. Biosci..

[18] Halliwell B. (1996). Commentary: Vitamin C: Antioxidant or Pro-Oxidant In Vivo?. Free Radic. Res. Commun..

[19] Molina N., Morandi A.C., Bolin A.P., Otton R. (2014). Comparative effect of fucoxanthin and vitamin C on oxidative and functional parameters of human lymphocytes. Int. Immunopharmacol..

[20] Dawood M.A.O., Koshio S. (2018). Vitamin C supplementation to optimize growth, health and stress resistance in aquatic animals. Rev. Aquac..

[21] NRC O.N. (1993). Nutrient Requirements of Fish.

[22] Wu F., Huang F., Wen H., Jiang M., Liu W., Tian J., Yang C.G. (2015). Vitamin C requirement of adult genetically improved farmed tilapia, *Oreochromis niloticus*. Aquac. Int..

[23] Biswas B.K., Biswas A., Junichi I., Kim Y.-S., Takii K. (2013). The optimal dietary level of ascorbic acid for juvenile Pacific bluefin tuna, *Thunnus orientalis*. Aquac. Int..

[24] Guary M., Kanazawa A., Tanaka N., Ceccaldi H.J. (1976). Nutritional Requirements of Prawn VI: Requirement for Ascorbic Acid. Mem. Fac. Fish Kagoshima Univ..

[25] Xie Z., Niu C., Zhang Z., Bao L. (2006). Dietary ascorbic acid may be necessary for enhancing the immune response in Siberian sturgeon (Acipenser baerii), a species capable of ascorbic acid biosynthesis. Comp. Biochem. Physiol. A Mol. Integr. Physiol..

[26] Ren T., Koshio S., Uyan O., Komilus C.F., Yokoyama S., Ishikawa M., Abdul M.K. (2008). Effects of Dietary Vitamin C on Blood Chemistry and Nonspecific Immune Response of Juvenile Red Sea Bream, *Pagrus major*. J. World Aquac. Soc..

[27] Shahkar E., Yun H., Kim D.-J., Kim S.-K., Lee B.I., Bai S.C. (2015). Effects of dietary vitamin C levels on tissue ascorbic acid concentration, hematology, non-specific immune response and gonad histology in broodstock Japanese eel, *Anguilla japonica*. Aquaculture.

[28] Tewary A., Patra B.C. (2008). Use of vitamin C as an immunostimulant. Effect on growth, nutritional quality, and immune response of *Labeo rohita* (Ham.). Fish Physiol. Biochem..

[29] Lin M.F., Shiau S.Y. (2005). Dietary L-ascorbic acid affects growth, nonspecific immune responses and disease resistance in juvenile grouper, *Epinephelus malabaricus*. Aquaculture.

[30] Ren T., Koshio S., Ishikawa M., Yokoyama S., Micheal F.R., Uyan O., Tung H.T. (2007). Influence of dietary vitamin C and bovine lactoferrin on blood chemistry and non-specific immune responses of Japanese eel, *Anguilla japonica*. Aquaculture.

[31] Kumari J., Sahoo P. (2006). Dietary immunostimulants influence specific immune response and resistance of healthy and immunocompromised Asian catfish *Clarias batrachus* to *Aeromonas hydrophila* infection. Dis. Aquat. Org..

[32] Barros M.M., Falcon D.R., Oliveira Orsi R., Pezzato L.E., Fernandes A.C., Guimaraes I.G., Fernandes A., Padovani C.R., Sartori M.M.P. (2014). Non-specific immune parameters and physiological response of Nile tilapia fed beta-glucan and vitamin C for different periods and submitted to stress and bacterial challenge. Fish Shellfish Immunol..

[33] Qiao J., Du Z., Zhang Y., Du H., Guo L., Zhong M., Cao J., Wang X. (2011). Proteomic identification of the related immune-enhancing proteins in shrimp *Litopenaeus vannamei* stimulated with vitamin C and Chinese herbs. Fish Shellfish Immunol..

[34] Trichet V.V., Santigosa E., Cochin E., Gabaudan J. (2015). The Effect of Vitamin C on Fish Health. Diet. Nutr. Addit. Fish Health.

[35] Xu H.J., Jiang W.D., Feng L., Liu Y., Wu P., Jiang J., Kuang S.Y., Tang L., Tang W.N., Zhang Y.A. (2016). Dietary vitamin C deficiency depresses the growth, head kidney and spleen immunity and structural integrity by regulating NF-kappaB, TOR, Nrf2, apoptosis and MLCK signaling in young grass carp (*Ctenopharyngodon idella*). Fish Shellfish Immunol..

[36] Xu H.J., Jiang W.D., Feng L., Liu Y., Wu P., Jiang J., Kuang S.Y., Tang L., Tang W.N., Zhang Y.A. (2016). Dietary vitamin C deficiency depressed the gill physical barriers and immune barriers referring to Nrf2, apoptosis, MLCK, NF-kappaB and TOR signaling in grass carp (*Ctenopharyngodon idella*) under infection of *Flavobacterium columnare*. Fish Shellfish Immunol..

[37] Mau A., Jha R. (2018). Aquaculture of two commercially important molluscs (abalone and limpet): Existing knowledge and future prospects. Rev. Aquac..

[38] Huang Z.-X., Chen Z.-S., Ke C.-H., Zhao J., You W.-W., Zhang J., Dong W.-T., Chen J. (2012). Pyrosequencing of *Haliotis diversicolor* Transcriptomes: Insights into Early Developmental Molluscan Gene Expression. PLoS ONE.

[39] Mai K. (1998). Comparative studies on the nutrition of two species of abalone, *Haliotis tuberculata* L. and *Haliotis discus hannai* Ino.: VII. Effects of dietary vitamin C on survival, growth and tissue concentration of ascorbic acid. Aquaculture.

[40] Wu C., Wang J., Xu W., Zhang W., Mai K. (2014). Dietary ascorbic acid modulates the expression profile of stress protein genes in hepatopancreas of adult Pacific abalone *Haliotis discus hannai* Ino. Fish Shellfish Immunol..

[41] Cai J., Han Y., Wang Z. (2006). Isolation of *Vibrio parahaemolyticus* from abalone (*Haliotis diversicolor* supertexta L.) postlarvae associated with mass mortalities. Aquaculture.

[42] Travers M.A., Le Goic N., Huchette S., Koken M., Paillard C. (2008). Summer immune depression associated with increased susceptibility of the European abalone, *Haliotis tuberculata* to *Vibrio harveyi* infection. Fish Shellfish Immunol..

[43] Loker E.S., Adema C.M., Zhang S.M., Kepler T.B. (2004). Invertebrate immune systems–not homogeneous, not simple, not well understood. Immunol. Rev..

[44] Mai K., Mercer J.P., Donlon J. (1995). Comparative studies on the nutrition of two species of abalone, *Haliotis tuberculata* L. and *Haliotis discus hannai* Ino. III. Response of abalone to various levels of dietary lipid. Aquaculture.

[45] Mai K., Mercer J.P., Donlon J. (1995). Comparative studies on the nutrition of two species of abalone, *Haliotis tuberculata* L. and *Haliotis discus hannai* Ino. IV. Optimum dietary protein level for growth. Aquaculture.

[46] Wu C., Zhang W., Mai K., Xu W., Zhong X. (2011). Effects of dietary zinc on gene expression of antioxidant enzymes and heat shock proteins in hepatopancreas of abalone *Haliotis discus hannai*. Comp. Biochem. Physiol. C. Toxicol. Pharm..

[47] AOAC (1995). Official Methods of Analysis.

[48] Anderson R.S., Brubacher L.L., Calvo L.R., Unger M.A., Burreson E.M. (2010). Effects of tributyltin and hypoxia on the progression of *Perkinsus marinus* infections and host defence mechanisms in oyster, *Crassostrea virginica* (Gmelin). J. Fish Dis..

[49] Xue J., Xu Y., Jin L., Liu G., Sun Y., Li S., Zhang J. (2008). Effects of traditional Chinese medicine on immune responses in abalone, *Haliotis discus hannai* Ino. Fish Shellfish Immunol..

[50] Góth L. (1991). A simple method for determination of serum catalase activity and revision of reference range. Clin. Chim. Acta.

[51] Schmedes A., Hlmer G. (1989). A new thiobarbituric acid (TBA) method for determining free malondialdehyde (MDA) and hydroperoxides selectively as a measure of lipid peroxidation. J. Am. Oil Chem. Soc..

[52] Currie K. (1991). GENORM: A generalized norm calculation. Comput. Geosci..

[53] Andersen C.L., Jensen J.L., Ørntoft T.F. (2018). Normalization of Real-Time Quantitative Reverse Transcription-PCR Data: A Model-Based Variance Estimation Approach to Identify Genes Suited for Normalization, Applied to Bladder and Colon Cancer Data Sets. Cancer Res..

[54] Livak K.J., Schmittgen T.D. (2001). Analysis of relative gene expression data using real-time quantitative PCR and the 2 (-Delta Delta C (T)) Method. Methods.

[55] Chen Y., Wang D., Peng H., Chen X., Han X., Yu J., Wang W., Liang L., Liu Z., Zheng Y. (2019). Epigenetically upregulated oncoprotein PLCE1 drives esophageal carcinoma angiogenesis and proliferation via activating the PI-PLCε-NF-κB signaling pathway and VEGF-C/Bcl-2 expression. Mol. Cancer.

[56] Ibiyo L.M.O., Atteh J.O., Omotosho J.S., Madu C.T. (2007). Vitamin C (ascorbic acid) requirements of *Heterobranchus longifilis* fingerlings. Afr. J. Biotechnol..

[57] Zou W., Lin Z., Huang Y., Limbu S.M., Rong H., Yu C., Lin F., Wen X. (2019). Effect of dietary vitamin C on growth performance, body composition and biochemical parameters of juvenile Chu’s croaker (*Nibea coibor*). Aquac. Nutr..

[58] Huang F., Wu F., Zhang S., Jiang M., Liu W., Tian J., Yang C., Wen H. (2017). Dietary vitamin C requirement of juvenile Chinese sucker (*Myxocyprinus asiaticus*). Aquac. Res..

[59] Xu Q., Luo K., Zhang S., Gao W., Zhang W., Wei Q. (2019). Sequence analysis and characterization of type I interferon and type II interferon from the critically endangered sturgeon species, *A. dabryanus* and *A. sinensis*. Fish Shellfish Immunol..

[60] Luo K., Di J., Han P., Zhang S., Xia L., Tian G., Zhanga W., Dun D., Xu Q., Wei Q. (2018). Transcriptome analysis of the critically endangered Dabry’s sturgeon (*Acipenser dabryanus*) head kidney response to *Aeromonas hydrophila*. Fish Shellfish Immunol..

[61] Esteban M.A. (2012). An Overview of the Immunological Defenses in Fish Skin. ISRN Immunol..

[62] Machałowski T., Jesionowski T. (2020). Hemolymph of molluscan origin: From biochemistry to modern biomaterials science. Appl. Phys. A.

[63] Dolashka P., Moshtanska V., Borisova V., Dolashki A., Stevanovic S., Dimanov T., Voelter W. (2011). Antimicrobial proline-rich peptides from the hemolymph of marine snail *Rapana venosa*. Peptides.

[64] Lee M.-H., Shiau S.-Y. (2002). Dietary vitamin C and its derivatives affect immune responses in grass shrimp, *Penaeus monodon*. Fish Shellfish Immunol..

[65] OrtuÑO J., Esteban M.A., Meseguer J. (1999). Effect of high dietary intake of vitamin C on non-specific immune response of gilthead seabream (*Sparus aurata* L.). Fish Shellfish Immunol..

[66] Abdo S.E., Gewaily M.S., Abo-Al-Ela H.G., Almeer R., Soliman A.A., Elkomy A.H., Dawood M.A.O. (2021). Vitamin C rescues inflammation, immunosuppression, and histopathological alterations induced by chlorpyrifos in Nile tilapia. Environ. Sci. Pollut. Res..

[67] Kong F., Zhu Y., Yu H., Wang X., Azm F.R.A., Yuan J., Tan Q. (2021). Effect of dietary vitamin C on the growth performance, nonspecific immunity and antioxidant ability of red swamp crayfish (*Procambarus clarkii*). Aquaculture.

[68] Rodrigues R.A., da Silva Nunes C., Fantini L.E., Kasai R.Y.D., Oliveira C.A.L., Hisano H., de Campos C.M. (2017). Dietary ascorbic acid influences the intestinal morphology and hematology of hybrid sorubim catfish (*Pseudoplatystoma reticulatum* × *P. corruscans*). Aquac. Int..

[69] Alexander J.B., Ingram G.A. (1992). Noncellular nonspecific defence mechanisms of fish. Annu. Rev. Fish. Dis..

[70] Holland M., Lambris J.D. (2002). The complement system in teleosts. Fish Shellfish Immunol..

[71] Wu T., Jiang Q., Wu D., Hu Y., Chen S., Ding T., Ye X., Liu D., Chen J. (2019). What is new in lysozyme research and its application in food industry? A review. Food Chem..

[72] Zhou Q., Wang L., Wang H., Xie F., Wang T. (2012). Effect of dietary vitamin C on the growth performance and innate immunity of juvenile cobia (*Rachycentron canadum*). Fish Shellfish Immunol..

[73] Kim J.-H., Kang J.-C. (2015). Influence of Dietary Ascorbic Acid on the Immune Responses of Juvenile Korean Rockfish *Sebastes schlegelii*. J. Aquat. Anim. Health.

[74] Yusuf A., Huang X., Chen N., Apraku A., Wang W., Cornel A., Rahman M.M. (2020). Impact of dietary vitamin c on plasma metabolites, antioxidant capacity and innate immunocompetence in juvenile largemouth bass, *Micropterus salmoides*. Aquac. Rep..

[75] Kawai T., Akira S. (2006). TLR signaling. Cell Death Differ..

[76] Kawasaki T., Kawai T. (2014). Toll-like receptor signaling pathways. Front. Immunol..

[77] Rauta P.R., Samanta M., Dash H.R., Nayak B., Das S. (2014). Toll-like receptors (TLRs) in aquatic animals: Signaling pathways, expressions and immune responses. Immunol. Lett..

[78] Schwabe R.F., Seki E., Brenner D.A. (2006). Toll-like receptor signaling in the liver. Gastroenterology.

[79] Takeuchi O., Akira S. (2010). Pattern Recognition Receptors and Inflammation. Cell.

[80] Akira S. (2003). Toll-like Receptor Signaling. J. Biol. Chem..

[81] Kawai T., Akira S. (2007). Signaling to NF-kappaB by Toll-like receptors. Trends Mol. Med..

[82] Sasai M., Yamamoto M. (2013). Pathogen Recognition Receptors: Ligands and Signaling Pathways by Toll-Like Receptors. Int. Rev. Immunol..

[83] O’Carroll S.J., Kho D.T., Wiltshire R., Nelson V., Rotimi O., Johnson R., Angel C.E., Graham E.S. (2015). Pro-inflammatory TNFα and IL-1β differentially regulate the inflammatory phenotype of brain microvascular endothelial cells. J. NeuroInflamm..

[84] Zhang J., Kong X., Zhou C., Li L., Nie G., Li X. (2014). Toll-like receptor recognition of bacteria in fish: Ligand specificity and signal pathways. Fish Shellfish Immunol..

[85] Sun J.-J., Xu S., He Z.-H., Shi X.-Z., Zhao X.-F., Wang J.-X. (2017). Activation of Toll Pathway Is Different between Kuruma Shrimp and *Drosophila*. Front. Immunol..

[86] Su M., Chen H., Wei C., Chen N., Wu W. (2014). Potential protection of vitamin C against liver-lesioned mice. Int. Immunopharmacol..

[87] Liang T., Chen X., Su M., Chen H., Lu G., Liang K. (2014). Vitamin C exerts beneficial hepatoprotection against Concanavalin A-induced immunological hepatic injury in mice through inhibition of NF-kappaB signal pathway. Food Funct..

[88] Yang L., Chu Y., Wang L., Wang Y., Zhao X., He W., Zhang P., Yang X., Liu X., Tian L. (2015). Overexpression of CRY1 protects against the development of atherosclerosis via the TLR/NF-kappaB pathway. Int. Immunopharmacol..

[89] Priyathilakaa T.T., Bathigeb S.D.N.K., Leea S., Namc B.-H., Lee J. (2019). Transcriptome-wide identification, functional characterization, and expression analysis of two novel invertebrate-type Toll-like receptors from disk abalone (*Haliotis discus discus*). Fish Shellfish Immunol..

[90] Takeshita F., Ishii K.J., Kobiyama K., Kojima Y., Coban C., Sasaki S., Ishii N., Klinman D.M., Okuda K., Akira S. (2005). TRAF4 acts as a silencer in TLR-mediated signaling through the association with TRAF6 and TRIF. Eur. J. Immunol..

[91] Viatour P., Merville M.P., Bours V., Chariot A. (2005). Phosphorylation of NF-kappaB and IkappaB proteins: Implications in cancer and inflammation. Trends Biochem. Sci..

[92] Bollrath J., Greten F.R. (2009). IKK/NF-kappaB and STAT3 pathways: Central signalling hubs in inflammation-mediated tumour promotion and metastasis. EMBO Rep..

[93] Masso-Silva J.A., Diamond G. (2014). Antimicrobial peptides from fish. Pharmaceuticals.

[94] Gerdol M., De Moro G., Manfrin C., Venier P., Pallavicini A. (2012). Big defensins and mytimacins, new AMP families of the Mediterranean mussel *Mytilus galloprovincialis*. Dev. Comp. Immunol..

[95] Hannemann N., Jordan J., Paul S., Reid S., Baenkler H.-W., Sonnewald S., Bäuerle T., Vera J., Schett G., Bozec A. (2017). The AP-1 Transcription Factor c-Jun Promotes Arthritis by Regulating Cyclooxygenase-2 and Arginase-1 Expression in Macrophages. J. Immunol..

[96] Monticelli L.A., Buck M.D., Flamar A.L., Saenz S.A., Tait Wojno E.D., Yudanin N.A., Osborne L.C., Hepworth M.R., Tran S.V., Rodewald H.R. (2016). Arginase 1 is an innate lymphoid-cell-intrinsic metabolic checkpoint controlling type 2 inflammation. Nat. Immunol..

[97] Hu Z., Wu B., Meng F., Zhou Z., Lu H., Zhao H. (2017). Impact of molecular hydrogen treatments on the innate immune activity and survival of zebrafish (Danio rerio) challenged with *Aeromonas hydrophila*. Fish Shellfish Immunol..

[98] Njus D., Kelley P.M., Tu Y.-J., Schlegel H.B. (2020). Ascorbic acid: The chemistry underlying its antioxidant properties. Free Radic. Biol. Med..

[99] Lushchak V.I. (2011). Environmentally induced oxidative stress in aquatic animals. Aquat. Toxicol..

[100] Chen Y.-J., Yuan R.-M., Liu Y.-J., Yang H.-J., Liang G.-Y., Tian L.-X. (2015). Dietary vitamin C requirement and its effects on tissue antioxidant capacity of juvenile largemouth bass, *Micropterus salmoides*. Aquaculture.

[101] Liang X.-P., Li Y., Hou Y.-M., Qiu H., Zhou Q.-C. (2017). Effect of dietary vitamin C on the growth performance, antioxidant ability and innate immunity of juvenile yellow catfish (*Pelteobagrus fulvidraco* Richardson). Aquac. Res..

[102] Hashimoto K. (2018). Essential Role of Keap1-Nrf2 Signaling in Mood Disorders: Overview and Future Perspective. Front. Pharm..

[103] Trenti A., Grumati P., Cusinato F., Orso G., Bonaldo P., Trevisi L. (2014). Cardiac glycoside ouabain induces autophagic cell death in non-small cell lung cancer cells via a JNK-dependent decrease of Bcl-2. Biochem. Pharmacol..

[104] Kim B.J., Ryu S.W., Song B.J. (2006). JNK- and p38 Kinase-mediated Phosphorylation of Bax Leads to Its Activation and Mitochondrial Translocation and to Apoptosis of Human Hepatoma HepG2 Cells. J. Biol. Chem..

[105] Borghetti G., Yamaguchi A.A., Aikawa J., Yamazaki R.K., de Brito G.A., Fernandes L.C. (2015). Fish oil administration mediates apoptosis of Walker 256 tumor cells by modulation of p53, Bcl-2, caspase-7 and caspase-3 protein expression. Lipids Health Dis..

[106] Vince J.E., Nardo D.D., Gao W., Vince A.J., Hall C., Mcarthur K., Simpson D., Vijayaraj S., Lindqvist L.M., Bouillet P. (2018). The Mitochondrial Apoptotic Effectors BAX/BAK Activate Caspase-3 and -7 to Trigger NLRP3 Inflammasome and Caspase-8 Driven IL-1β Activation. Cell Rep..

[107] Cheng C.-H., Liang H.-Y., Luo S.-W., Wang A.-L., Ye C.-X. (2018). The protective effects of vitamin C on apoptosis, DNA damage and proteome of pufferfish (*Takifugu obscurus*) under low temperature stress. J. Biol..

[108] Feidantsis K., Georgoulis I., Giantsis I.A., Michaelidis B. (2021). Treatment with ascorbic acid normalizes the aerobic capacity, antioxidant defence, and cell death pathways in thermally stressed *Mytilus galloprovincialis*. Comp. Biochem. Physiol. Part B Biochem. Mol. Biol..

