# Supplementary material for: Ascorbic Acid Regulates the Immunity, Anti-Oxidation and Apoptosis in Abalone Haliotis discus hannai Ino
Source: Antioxidants (Basel). 2021 Sep 13;10(9):1449. doi: 10.3390/antiox10091449 (PMC8465606; doi:10.3390/antiox10091449)
Supplement: Supplementary file 1 [file antioxidants-10-01449-s001.zip › antioxidants-1340961-supplementary.pdf]

Supplementary Materials:

Supplementary Table S1. Primer used in this study for qPCR

Target gene	Forward primer (5'→3')	Reverse primer (5'→3')	Accession number
tlr2	CCTGGAGCAAATCATTCCCA	ATTGCCAAGATCTAAGCTTCGT	MT919299
tlr4	GGACGCCGACATCAGACACAT	CAGCCACTTCAGAGACGACAGAG	MT919300
tlr-a	CATCAAACTACCTCGGAGAGC	AGAACCACCAGCATTAGCAT	MH205666
tlr-b	ACGATGAACTTGTTTTGCACT	GTTTCTGCTTCCATCTAGCCTT	MH205667
myd88	TTGCCCTACACAATGCGAGT	CCTCCGGTCAATGATACGTTC	KU351389.1
tram	GGTACGGCTTGGCTCCATAACTC	GTCTTCTTGGCAGCAGCACTCTT	MW889876
irak4	TGCAACTGCCAGATTAGCACCT	TGACGTGCCGATCACAACCA	KU351646.1
traf6	TGCCTGCTTATCCTGCGAGAAC	TCACAGCCTTGCCTTGAGTTGG	MW889877
traf4	CAACCGTGCCATGTCCGTTCA	CCGTCTGTTACCTGCGTCACTG	MW889878
ikkα	ACATGTCCAGACGCCCAA	CCAAGACCAACAAGCACAC	MT919293
iκbα	GGAGGGACAGAGGGAGGATT	GACCCGTCCACCATGTCATT	JX235971.1
p38 mapk	ATGGGAGCGTATGGGCAAGTGT	TGGCATGGATGGCTGTCTGGAA	MW889879
jnk	CCATCGGTTCAGGTGCTCAAGG	GCGTGTGTGACATTCTGGAAGG	MT482023
nf-κb	TGCTGGTTGTATGTGGGAGGAC	GAGGTCTCGTTGTCGGATGGTC	GQ903763.1
ap-1	TGTGTCGTCTGCTTCACGTT	TGTGGCTCCTCTTTCAACCG	GU186850.1
tnf-α	TGGACTCAATGGAACGACAGG	GCCCTTCTCATACCGCATCC	EU863217.1
il 16	GGAAGATGGTGACGACTGGTGAG	GCTGTAGACGCTGGTCCTTCTC	MW889880
β-defensin	CGTCACCTGCGACCTGCTG	CAGAATCACCGAAGGAGTTGCC	FJ864724.1
mytimacin 6	ACAATGTTCCTCGCTATCGTCGT	TCATCCAGCAGTCAGAAGCATCC	MF066909.1
arginase I	CGTACTACATAGCTGAGGAGGTG	CCATAGAAACGGGACATGACTT	MT919298
nrf2	CGACAGCAGCAGCAGCAATG	GGTGAGGACATCTGGAGGCATC	MT919295
keap1	CGACCATGTCCACTAACCGTATTG	TAGCAGTCCATAGCGATAACACCAG	MT919296
gpx	TTTCGACCGTCCAACTTGCT	CAAAGTTCCAGTACACGTCCC	GU254066.1
cat	GGAACCCTTTCGACCTCACC	ATCTGTTTCCACGTCAGCAA	DQ821496.1
gst	GTTTGCCTTCTACTCGACGGTA	TCCGTTGTTGTCGCCTCC	DQ530212.1
cuznsod	CTTCAACCCCTTTCGGCAAG	ACCAGCATGTCTGTTTTCGT	DQ530214.1
bax	CGGAGCACATTTCTCAACGTA	AAGTAAAAGAGGGCAACCAC	MT501464
caspase3	AAATTCTTGAAACCGCAGACAC	ACCAACAAGTGCGATACAGT	FJ864720.1
caspase7	TGAAGAAATACAGCACGACCA	TACTACGGCCATCAATCTGGG	MT919294
bcl-2	TGGTCGCTTTGTTTACTTTCGG	AGGGTTCTCGTTTCGCTTCTC	MT482025
β-actin	ACTCCATCATGAAGTGCGAT	TTCTGCATACGGTCAGCGAT	AY380809.1
gapdh	ACGCCTCCTGCACAACCAA	ACAATGCCGAAGTTGTCGTT	EF103374.1

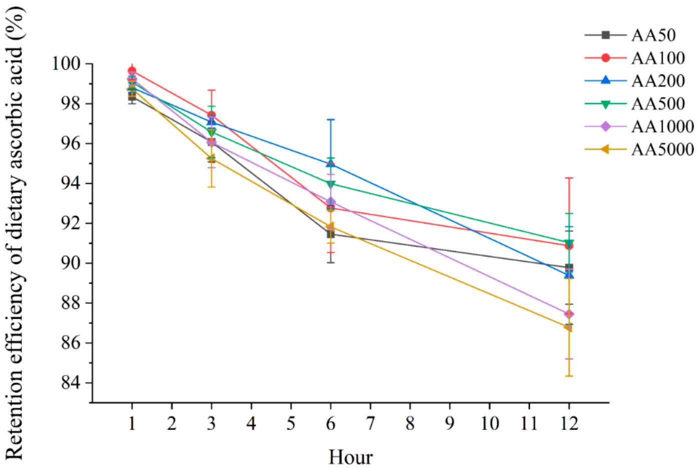

Supplementary Figure S1. Retention efficiency of dietary ascorbic acid in experimental diets at different intervals immersed in seawater. Data were expressed as Mean ± SE (n = 3).
